# Dissecting the major genetic components underlying cotton lint development

**DOI:** 10.1093/genetics/iyad219

**Published:** 2023-12-26

**Authors:** Yali Sun, Yuman Yuan, Shoupu He, Warwick Stiller, Iain Wilson, Xiongming Du, Qian-Hao Zhu

**Affiliations:** National Key Laboratory of Cotton Bio-Breeding and Integrated Utilization, Institute of Cotton Research, Chinese Academy of Agricultural Sciences, Anyang, Henan 455000, China; College of Agriculture, Shanxi Agricultural University, Taigu, Shanxi 030801, China; CSIRO Agriculture and Food, GPO Box 1700, Canberra, ACT 2601, Australia; National Key Laboratory of Cotton Bio-Breeding and Integrated Utilization, Institute of Cotton Research, Chinese Academy of Agricultural Sciences, Anyang, Henan 455000, China; CSIRO Agriculture and Food, Locked Bag 59, Narrabri, NSW 2390, Australia; CSIRO Agriculture and Food, GPO Box 1700, Canberra, ACT 2601, Australia; National Key Laboratory of Cotton Bio-Breeding and Integrated Utilization, Institute of Cotton Research, Chinese Academy of Agricultural Sciences, Anyang, Henan 455000, China; CSIRO Agriculture and Food, GPO Box 1700, Canberra, ACT 2601, Australia

**Keywords:** lint percentage, lintless, fuzzless, bulk segregant analysis, haplotype, allele pyramiding

## Abstract

Numerous genetic loci and several functionally characterized genes have been linked to determination of lint percentage (lint%), one of the most important cotton yield components, but we still know little about the major genetic components underlying lint%. Here, we first linked the genetic loci containing *MYB25-like_At* and *HD1_At* to the fiberless seed trait of ‘SL1-7-1’ and found that *MYB25-like_At* and *HD1_At* were very lowly expressed in ‘SL1-7-1’ ovules during fiber initiation. We then dissected the genetic components involved in determination of lint% using segregating populations derived from crosses of fuzzless mutants and intermediate segregants with different lint%, which not only confirmed the *HD1_At* locus but identified the *HD1_Dt* locus as being the major genetic components contributing to fiber initiation and lint%. The segregating populations also allowed us to evaluate the relative contributions of *MYB25-like_At*, *MYB25-like_Dt*, *HD1_At*, and *HD1_Dt* to lint%. Haplotype analysis of an Upland cotton (*Gossypium hirsutum*) population with 723 accessions (including 81 fuzzless seed accessions) showed that lint% of the accessions with the LP allele (higher lint%) at *MYB25-like_At*, *MYB25-like_Dt*, or *HD1_At* was significantly higher than that with the lp allele (lower lint%). The lint% of the Upland cotton accessions with 3 or 4 LP alleles at *MYB25-like* and *HD1* was significantly higher than that with 2 LP alleles. The results prompted us to propose a strategy for breeding high-yielding cotton varieties, i.e. pyramiding the LP alleles of *MYB25-like* and *HD1* with new lint% LP alleles without negative impact on seed size and fiber quality.

## Introduction

Cotton seeds produce long (lint) and short (fuzz) types of fibers. Both are single-celled and arise from the epidermal cells of the seed coat. The identity of lint fibers is determined at least 1 day before flowering and lint fiber initials start their outgrowth on the day of flowering (Qin *et al*. 2022). After ∼25 days of elongation, lint fibers approach their maximum length, which can be as long as ∼35 mm in Upland cotton (*Gossypium hirsutum* L.), and become mature at ∼55 days-post-anthesis (dpa) (Kim and Triplett 2001). Fuzz fibers initiate at ∼3 dpa and do not elongate to the same extent as the lint fibers and usually have a length of <5 mm (Joshi *et al*. 1967; Stewart 1975). Upland cotton fuzzless mutants and most Pima, Egyptian and Sea Island cotton (*Gossypium barbadense* L.) accessions produce seeds without fuzz fibers (Fang *et al*. 2018). While fiberless (lintless and fuzzless) cotton mutants have been well documented, no mutant bearing only fuzz fibers has ever been reported, thanks to the expression profile of the 2 *MYB25-like* homeologs during fiber initiation (Zhu *et al*. 2018).

Producing a high yield and high quality of lint fibers is the main goal of global cotton production despite the fuzz fibers (used for making specific paper and cellulose products) and cotton seeds (used for producing cooking oil and as a cattle feed supplement) also being valuable natural resources. Lint percentage (lint%) is one of the most important fiber yield components and is commonly used as a proxy for lint yield during breeding because of its high heritability. Increasing lint% is thus one of the major strategies adopted by cotton breeders for improving lint yield of cotton varieties. As a result, newly released cotton varieties usually have a higher lint% than obsolete or superseded ones (Ma *et al*. 2019; Conaty and Constable 2020). Approximately a quarter to a third of the cotton seed epidermal cells differentiate into fiber initials and eventually become lint fibers (Lang 1938; Stewart 1975). Turning more seed epidermal cells into lint fiber initials and/or repressing the development of fuzz fibers are therefore anticipated to increase the yield of lint fibers. To that end, it demands a deeper understanding the genetic and molecular basis underlying differentiation and determination of lint and fuzz fibers. Fiber mutants, including fuzzless and fiberless mutants, are indispensable genetic resource for achieving that goal.

Several genetic loci, including dominant *N_1_* and *N_5_* and recessive *n_2_*, *n_3_*, and *n_4_^t^*, contributing to fuzzless cotton seeds have been documented (Bechere *et al*. 2012; Fang *et al*. 2018; Zhu, Stiller, *et al*. 2021). And *fl1* (*fibreless1*) was proposed by Turley and Kloth (2008) to be the locus contributing to the fiberless phenotype of ‘SL1-7-1’. The fuzzless phenotype of the *N_1_* mutant is caused by loss-of-function of *MYB25-like_At* (At representing the A-subgenome of the tetraploid cotton) thanks to short interfering RNAs generated from the 3′ portion of the gene due to the presence of natural antisense transcripts (Wan *et al*. 2016). The gene underlying the *n_2_* mutation is likely to be the Dt-subgenome *MYB25-like_Dt* based on genetic mapping of the recessive fuzzless trait of *G. barbadense* and comparison of the expression profiles of *MYB25-like_At* and *MYB25-like_Dt* during fiber initiation in cotton accessions with different fiber phenotypes (Zhu *et al*. 2018). The recessive *n_3_* locus was inferred based on observations of its involvement in the full expression of the fuzzless seed phenotype in the homozygous *n_2_* genetic background (Turley and Kloth 2002) and has recently been proposed to be an allele of *N_1_* (Chen *et al*. 2020). Seeds of the *n_4_^t^* and *N_5_* mutants are fuzzless but with a tuft of short fibers attached to the micropyle end (so-called tufted trait) (Bechere *et al*. 2012; Zhu, Stiller, *et al*. 2021). The *n_4_^t^* locus has been mapped to an ∼411-kb genetic interval on Chr-D04 but the responsible gene and mutation are yet to be identified (Naoumkina *et al*. 2021). The candidate mutation underpinning the dominant fuzzless-tufted seed phenotype of the *N_5_* mutant has been located to an ∼250-kb genomic region on Chr-D13. The interval contains a couple of genes expressing significantly differently between the near-isogenic lines showing the mutant and wild-type seed phenotypes, thereby they were proposed to be the candidate genes (Zhu, Stiller, *et al*. 2021).

Four natural fiberless mutants have been reported, including ‘L40’ (Musaev and Abzalov 1972), fuzzless-lintless ‘MCU-5’ (Nadarajan and Rangasamy 1988), ‘Xu142fl’ (Zhang and Pan 1991), and ‘SL1-7-1’ (Turley and Kloth 2008). A fifth fiberless mutant (‘MD17’) was found among the progeny from the cross between the dominant (*N_1_*) and the recessive (*n_2_*) fuzzless mutants (Turley 2002). Of these fiberless mutants, ‘Xu142fl’ is the one that has been used in many genetic and molecular studies to explore the mechanism underpinning fiber initiation. Apart from *n_2_* and *n_3_*, ‘Xu142fl’ was proposed to contain a recessive lintless mutation, *li_3_* (Zhang and Pan 1991). The identity of *Li_3_* is still controversial because 2 MYB-MIXTA-like (MML) transcription factors (*MYB25-like_Dt* and *GhMML4_Dt*) located next to each other on Chr-D12 have been proposed to be the corresponding gene. Wu *et al*. (2018) proposed *GhMML4_Dt* being *Li_3_* based on genetic mapping using segregating populations derived from cross between the *n_2_* fuzzless mutant and ‘Xu142fl’ and the presence of a single nucleotide polymorphism (SNP) that induces a stop codon (TAA) in the 3rd exon of *GhMML4_Dt* in ‘Xu142fl’. Using the same genetic materials, a later study mapped the *li_3_* mutation to the same region as that reported by Wu *et al*. (2018) but found that the stop codon is also present in *GhMML4_Dt* of *G. hirsutum* accessions with normal fibers, and instead, a retrotransposon insertion was found in the 2nd exon of *MYB25-like_Dt* in ‘Xu142fl’ so *MYB25-like_Dt* was proposed to be *Li_3_* (Chen *et al*. 2020). ‘SL1-7-1’ was proposed to have 3 mutations, a dominant (*N_1_*) and a recessive (*n_3_*) mutation related to fuzz development and a recessive (*fl_1_*) mutation related to lint development (Turley and Kloth 2008). Given that *n_3_* was proposed to be allelic to *N_1_* (Chen *et al*. 2020), further investigation is required for the mutations underlying the fiberless phenotype of ‘SL1-7-1’.

The fuzzless alleles have variable degrees of negative impact on the development of lint fibers, and consequently on lint%. Cotton accessions containing homozygous *N_1_* have a lint% ranging from 0.7 to 23.6%, lower than homozygous *n_2_* accessions (24.4%) and normal fuzzy seeded accessions (37.7%) (Turley *et al*. 2007). The higher the number of fuzzless alleles, the lower the lint% (Turley and Kloth 2008) implies an additive effect of the fuzzless alleles on lint development. Compared to *N_1_* and *n_2_*, the fuzzless-tufted alleles *n_4_^t^* and *N_5_* have a weaker negative impact on lint development and there is no significant penalty on lint% and lint yield in the lines containing either mutation. These alleles thus have the potential to be used in breeding fuzzless elite Upland cotton varieties to reduce energy consumption during ginning (Bechere and Auld 2014; Zhu, Stiller, *et al*. 2021).

Apart from the fuzzless mutations mentioned above, many other genetic loci have been identified to be associated with lint% based on mapping of quantitative trait loci (QTL) and genome-wide association studies (GWAS) in *G. hirsutum* and *G. barbadense* (Fang *et al*. 2017; Ma *et al*. 2018, 2019; Yu *et al*. 2021; Chen *et al*. 2022; Zhao *et al*. 2022; Li *et al*. 2023). For instance, a GWAS with 258 Upland cotton accessions identified 71 genetic loci associated with yield traits, including 9 lint% related loci (Fang *et al*. 2017); and in a GWAS with 336 *G. barbadense* accessions, a Chr-A05 locus was found to be strongly associated with lint% and down-regulating the potential candidate gene (*Gbar_A05G014160* or *GbLP1*) by virus-induced gene silencing decreases lint% (Zhao *et al*. 2022). While some of the candidate genes identified by GWAS to be associated with lint% could affect the trait by regulating fiber initiation since they have a higher expression level in 0–5 dpa ovules (Ma *et al*. 2018; Zhao *et al*. 2022), some might affect lint% through their involvement in fiber elongation and/or development of the lint fiber secondary cell wall (Ma *et al*. 2019; Yu *et al*. 2021; Zhao *et al*. 2022). Furthermore, most genetic loci associated with lint% have pleiotropic negative effects on other agronomic traits (Fang *et al*. 2017; Li *et al*. 2023). For instance, 2 high lint% loci on Chr-D03 negatively contribute to fiber fineness or boll number per plant (Li *et al*. 2023).

*GhHD1* is a homeodomain-leucine zipper (HD-Zip) transcription factor (TF) expressed predominantly in epidermal tissues during the fiber initiation stage (Walford *et al*. 2012; Qin *et al*. 2022). Silencing *GhHD1* delayed the timing of fiber initiation and overexpressing *GhHD1* increased the number of fiber initials on the seed surface (Walford *et al*. 2012). The results of a recent single-cell transcriptome study suggest that *GhHD1* works in concert with *MYB25-like* and other TFs in regulating fiber differentiation, initiation, and rapid elongation during the fiber initiation and early developmental stage (Qin *et al*. 2022). A retrotransposon insertion in the 9th exon of *HD1_At* in *G. barbadense* results in loss-of-function of the gene and the glabrous stem phenotype (Ding *et al*. 2015; Niu *et al*. 2019), although the link between this mutation and the low lint% of *G. barbadense* is yet to be established. But in *Gossypium arboreum*, a cultivated diploid cotton species, the fiberless seeds and glabrous stem observed in the *SMA-4* mutant (Beasley and Egli 1977) has been suggested to be linked to an alternative splicing mutation in *GaHD1* (Ding *et al*. 2020).

To determine the major genetic components regulating lint fiber initiation and lint% as a guide to breeding higher yielding cotton varieties, in this study, we mapped the genetic loci responsible for the fiberless seed trait of ‘SL1-7-1’ and dissected, in a stepwise procedure, the genetic loci associated with fiberless seeds and lint% using segregating populations derived from backcrosses or cross between sibling segregants with extremely different lint%. We found that the genetic regions containing *MYB25-like_At* and *HD1_At* are associated with the fiberless trait of ‘SL1-7-1’ and that the genetic region harboring *HD1_Dt* is also associated with lint%. The relative contributions of the *MYB25-like_At*, *MYB25-like_Dt*, *HD1_At*, and *HD1_Dt* loci to lint% was further evaluated in different biparental segregating populations and a diversity panel including both *G. hirsutum* and *G. barbadense* cotton accessions. Finally, we propose genetic solutions for lint% improvement.

## Materials and methods

### Plant materials

Two sets of cotton materials were used, 1 for biparental crosses to analyze lint% segregation (done in Australia), another (a diversity panel) for association and haplotype analyses (done in China).

**Fig. 1.**
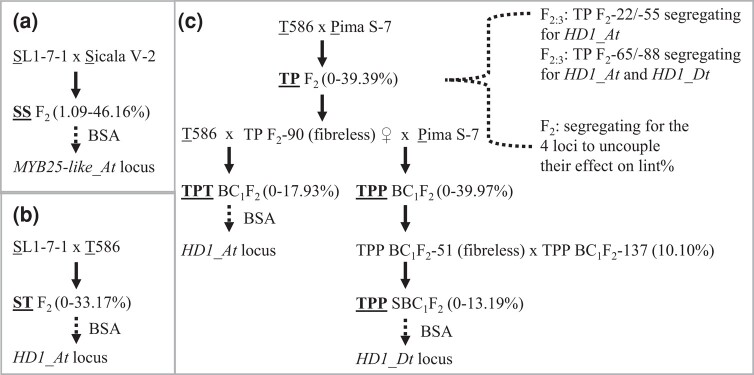
Overview of the biparental segregating populations used in uncoupling the genetic loci contributing to lint development. a) Bulk segregant analysis (BSA) of the SS F_2_ population (‘SL1-7-1’ × ‘Sicala V-2’) identified the *MYB25-like_At* locus to be associated with lower lint percentage of ‘SL1-7-1’. b) BSA of the ST F_2_ population (‘SL1-7-1’ and ‘T586’; both with dysfunctional *MYB25-like_At*) linked the *HD1_At* locus of ‘SL1-7-1’ to lower lint percentage. c) The fiberless segregant (TP F_2_-90) derived from ‘T586’ × ‘Pima S-7’ (‘Pima S-7’ is assumed to be dysfunctional at the *MYB25-like_Dt* locus) was backcrossed to ‘T586’ or ‘Pima S-7’ to uncover additional loci associated with lint percentage in addition to *MYB25-like_At* and *MYB25-like_Dt*. 4 F_2:3_ populations from TP F_2_ segregating for only *HD1_At* or both *HD1_At* and *HD1_Dt* were used to assess the impact of *HD1* on lint development. Meanwhile, TP F_2_ was used to investigate the impact of *MYB25-like_At*, *MYB25-like_Dt*, *HD1_At*, and *HD1_Dt* on lint percentage.

Four cotton accessions were used in biparental crosses, including 3 Upland cotton (*G. hirsutum*) (‘Sicala V-2’, ‘T586’, and ‘SL1-7-1’) and 1 *G. barbadense* accession (‘Pima S-7’) (Fig. 1). ‘Sicala V-2’ (lint% 42.13%) is an obsolete commercial Australian cultivar released by CSIRO in 1994. ‘T586’ is a genetic standard line containing several dominant mutations including the fuzzless *N_1_* mutation and shows a fuzzless-linted phenotype (lint% 11.33%). ‘SL1-7-1’ is a fiberless (lintless and fuzzless) mutant and produces a very small amount of fiber (lint% 1.01%) under the glasshouse conditions in Canberra, Australia (Supplementary Fig. 1). Seeds of ‘Pima S-7’, an obsolete commercial American cultivar (lint% 34.36%), are fuzzless, presumably due to the presence of the recessive fuzzless *n_2_* mutation (Zhu *et al*. 2018). ‘T586’, ‘SL1-7-1’, ‘Pima S-7’, and the genetic standard line of Upland cotton ‘TM-1’ (lint% 35.50%) were used in gene expression analysis.

The diversity panel included 723 *G. hirsutum* accessions (including 642 fuzzy seeded accessions and 81 fuzzless seed ones) and 326 *G. barbadense* accessions. These cotton accessions were selected, based on the rationale of maximizing genetic diversity, from the cotton germplasm collection at the National Midterm Genetic Bank of Cotton at the Institute of Cotton Research of the Chinese Academy of Agricultural Sciences, Anyang, China.

### Growth conditions

In Canberra, Australia, cotton plants were grown in glasshouses at 28 ± 2°C with natural lighting. Each plant was grown in a pot with a diameter of 23 cm. For each segregating population, the parental lines were grown alongside the segregants.

In China, the *G. hirsutum* accessions were grown in the field of the Experimental Station of the Institute of Cotton Research of the Chinese Academy of Agricultural Sciences in 2017 and 2018. The *G. barbadense* accessions were grown in the field in Sanya, Hainan in 2014 and in Aksu, Xinjiang in 2015 and 2016. All experiments were conducted with a randomized design with 3 replicates. Each replicate consisted of 3 8-m rows with a row space of 80 cm. Cotton field management was carried out by following the local requirements for commercial cotton production.

### Phenotyping

For the segregating populations generated by biparental crosses, the phenotypes of interest were fiberless seeds and lint%. Seed cotton harvested from the segregants of each segregating population was first observed for seed fiber phenotype to separate them into 2 groups—fiberless and fiber-bearing. Fiber-bearing seed cottons were hand-ginned and lint% was calculated using the formula:


$$\mathrm{Lint}\text{\%}=\frac{\mathrm{weight}\;\mathrm{of}\;\mathrm{harvested}\;\mathrm{lint}}{\mathrm{weight}\;\mathrm{of}\;\mathrm{unginned}\;\mathrm{seed}\;\mathrm{cotton}}\;\times 100.$$

For the diversity panel used in association and haplotype analyses, based on “The Standardised Data Collection and Description Protocol for Evaluation of Cotton Germplasm” of China, 30 open bolls per plot were collected from the middle fruiting branches of cotton plants and ginned by a roller ginning machine after drying to calculate lint% using the aforementioned formula. BLUPs (best linear unbiased predictors) of lint% were estimated using LME4 of the R package and used in comparison.

### DNA sample preparation, bulk segregant analysis, and genotyping

For the plants used in segregation analysis in Australia, DNA extraction and KASP (Kompetitive Allele-Specific PCR) genotyping were performed as described previously (Zhu *et al*. 2016). For each bulk segregant analysis (BSA; Zhu *et al*. 2017), 2 DNA pools were prepared using the segregants of the corresponding segregating population with the lowest (or fiberless) and highest lint% (Supplementary File 1). DNA of the segregants was individually extracted and an equal amount of DNA from each segregant was taken and pooled for whole-genome shot-gun sequencing through Azenta (Indianapolis, USA). Sequencing was done using the Illumina HiSeq2500 in the format of 150-bp paired-end reads with a depth of ∼25× coverage. Read mapping, single nucleotide polymorphism (SNP) call and SNP frequency distribution plotting were done by following the approaches reported previously (Zhu *et al*. 2018). The in-house genome of ‘Sicala V-2’, generated by mapping ‘Sicala V-2’ reads to the ‘TM-1’ reference genome (Wang *et al*. 2019), was used as the reference for BSA of the SS F_2_ population, and the in-house genome of ‘T586’, generated by mapping ‘T586’ reads to the same ‘TM-1’ reference genome, was used as the reference for BSA of ST F_2_, TPT BC_1_F_2_, and TPP SBC_1_F_2_ (Fig. 1). The genomic region(s) showed the most divergent SNP frequency in the 2 pools and with a frequency > 0.8 in 1 pool and ∼0.5 in another pool were considered as the candidate region(s) associated with lint%. The SNP frequency distribution plots of SS F_2_ and ST F_2_ were generated using a 1-Mbp sliding window and the plots of TPT BC_1_F_2_ and TPP SBC_1_F_2_ were generated using a 500-kb sliding window.

KASP was done on the ViiA7 Real-Time PCR system (Life Technologies, California, USA) using the KASP master mix sourced from LGC Group (Middlesex, UK). All individuals of each segregating population were used in KASP genotyping. The KASP markers used to narrow down the genomic region containing the causative locus associated with lint% were designed for the region defined by BSA. The sequences of these KASP markers together with those used in defining the genotype of the *MYB25-like_At*, *MYB25-like_Dt*, *HD1_At*, and *HD1_Dt* loci in different segregating populations are shown in Supplementary Table 1.

For the diversity panel grown in China, CTAB was used in isolating DNA from cotton seedlings germinated under growth cabinet conditions (18 hours light/6 hours dark, T_m_ 26°C, and relative humidity 65%). Genotyping of the diversity panel was done based on whole-genome resequencing data (∼10× coverage) generated by Illumina sequencing as previously described (He *et al*. 2021; Wang *et al*. 2022). The fuzzy seeded *G. hirsutum* and the *G. barbadense* accessions have been reported previously (He *et al*. 2021; Wang *et al*. 2022) and the fuzzless *G. hirsutum* accessions were used for the first time in this study. After quality control, the resequencing data of each accession were aligned to the ‘TM-1’ reference genome (Yang *et al*. 2019) with SNP calling using the Unified Genotyper module of the Genome Analysis Toolkit (V3.1, Mckenna *et al*. 2010) based on the default parameters. The SNPs that met the following criteria were kept for further use: minor allele frequency ≥ 0.05, coverage depth ≥ 3 and ≤ 50, quality score ≥ 20, and max-missing rate ≤ 20%.

### Haplotype analysis of *MYB25-like* and *HD1*

Our focus was on the 4 loci containing *MYB25-like_At*, *MYB25-like_Dt*, *HD1_At*, or *HD1_Dt*, so for each locus, the SNPs that were retained based on the criteria aforementioned and are within each of the annotated gene and its 2-kb flanking region were used in haplotype analysis. The SNPs were used to construct the NJ phylogenetic tree of the cotton accessions by using Archaeopteryx in TASSEL 5.2.51 (Bradbury *et al*. 2007) and then the accessions were separated into 3 groups based on their haplotypes, i.e. LP (associated with higher lint%), lp (associated with lower lint%), and heterozygous. The statistical significance for the association of the LP and lp haplotypes with lint% was carried out by the Mann–Whitney test in Graphpad Prism 8 (version 8.4.3; https://www.graphpad.com/). The haplotype heatmaps were ordered according to the local genotype clustering by Fig Tree (version 1.4.4; http://tree.bio.ed.ac.uk/software/figtree).

### Analysis of the effect of allelic/haplotype combination on lint percentage

To investigate the effect of different combinations of haplotypes of the 4 genes (*MYB25-like_At*, *MYB25-like_Dt*, *HD1_At*, and *HD1_Dt*) on lint% in *G. hirsutum*, we separated the 723 *G. hirsutum* accessions into fuzzless and fuzzy seeded 2 groups and then the fuzzy seeded accessions were further grouped based on the number of LP haplotypes at the 4 loci and compared their mean lint% values (BLUPs).

### RNA sample preparation and gene expression analysis

Whole ovules were collected from ‘Pima S-7’, ‘TM-1’, ‘T586’, and ‘SL1-7-1’ at −1, 0, 1, 3, 5, and 7 dpa and used in RNA extraction. Total RNA was isolated using the Maxwell RSC Plant RNA Kit (Promega, Madison, USA) by following the manufacture's instruction. Quantitative real-time PCR (qRT-PCR) was carried out as previously described (Zhu *et al*. 2018) on the ViiA7 Real-Time PCR System (Life Technologies, California, USA) using the FastStart Universal SYBR Green Master Mix (ROX) (Roche, Basel, Switzerland). The expression levels were determined based on 3 biological replicates each with 3 technical replicates. The cotton ubiquitin gene (GenBank accession no. EU604080) was used as the reference gene for calculation of the relative expression level of individual genes in each sample based on the formula of −2^ΔCt^. Because primers specific to *MYB25-like_At* could not be confidently designed, the total expression level of *MYB25-like_At* and *MYB25-like_Dt* and *MYB25-like_Dt* alone was measured. Primers used in qRT-PCR are provided in Supplementary Table 1. All primer pairs had a similar PCR efficiency (89.9–99.2%) determined by LinRegPCR (http://www.hartfaalcentrum.nl/index.php?main=files&sub=LinRegPCR).

## Results

### The fiberless trait of ‘SL1-7-1’ is associated with the loci containing dysfunctional *MYB25-like_At* and *HD1_At* genes

**Fig. 2.**
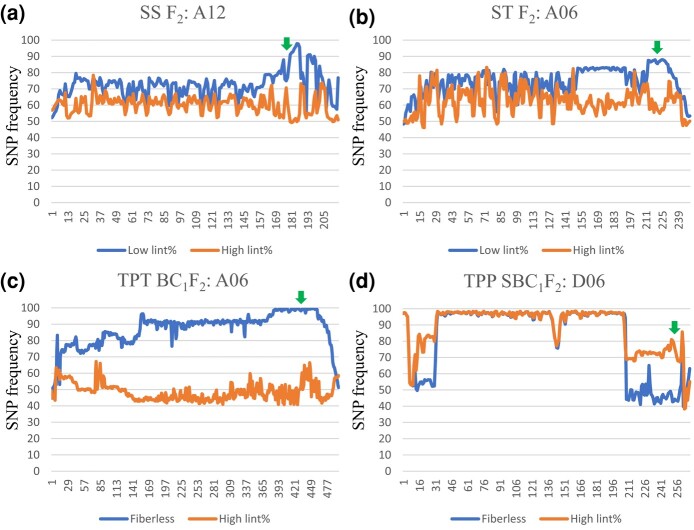
The genomic regions (indicated by green arrow heads) identified to be associated with lint percentage in 4 populations based on bulk segregant analysis (BSA) using single nucleotide polymorphism (SNP). SNP distribution plots across all chromosomes are provided in Supplementary Figs. 2,3,6, and 8. a) The region containing *MYB25-like_At* was found to be associated with lint development in SS (‘SL1-7-1’ × ‘Sicala V-2’). SNPs were called based on BSA reads mapped to the in-house ‘Sicala V-2’ genome sequence. b and c) The region containing *HD1-At* was identified to be associated with lint percentage in ST F_2_ (‘SL1-7-1’ × ‘T586’) and TPT BC_1_F_2_ [(‘T586’ × ‘Pima S-7’) × ‘T586’]. SNPs were called based on BSA reads mapped to the in-house ‘T586’ genome sequence. d) The genomic region containing *HD1-Dt* was identified to be associated with lint percentage in TPP SBC_1_F_2_ [(‘T586’ × ‘Pima S-7’) × ‘Pima S-7’] (fiberless) × [(‘T586’ × ‘Pima S-7’) × ‘Pima S-7’] (lint percentage: 10.10%). SNPs were called based on BSA reads mapped to the in-house ‘T586’ genome sequence. The numbers on the *X*-axis represent genomic bins with 1 Mbp (SS F_2_ and ST F_2_) or 500 kb (TPT BC_1_F_2_ and TPP SBC_1_F_2_) overlapping between the adjacent bins.

To investigate the genetic basis of the fiberless trait of ‘SL1-7-1’, an F_2_ population (SS F_2_ with 108 plants) was generated by crossing ‘SL1-7-1’ to ‘Sicala V-2’, a linted and fuzzy seeded variety (Fig. 1). Of the 108 F_2_ segregants, 29 produced fuzzy seeds with a lint% of 34.50–46.16% and 79 produced fuzzless seeds with a lint% of 1.09–40.93%. From the 79 fuzzless-seed F_2_ plants, 2 DNA pools were made for bulk segregant analysis (BSA). One included 15 plants with a lint% < 10% (1.09–9.42%) and the other included 15 plants with a lint% > 38% (38.07–40.93%). Single nucleotide polymorphism (SNP) calls were done after aligning the sequence reads to the in-house ‘Sicala V-2’ genome sequence. Based on the distribution of SNP frequency across all 26 chromosomes, a ∼3.5-Mbp genomic region on Chr-A12 was identified to be the best candidate region associated with lint% (Fig. 2a; Supplementary Fig. 2). The region contains *MYB25-like_At*, which functions as a master regulator of lint and fuzz development (Walford *et al*. 2011; Zhu *et al*. 2018). The dominant fuzzless mutant *N_1_* is caused by loss-of-function of *MYB25-like_At* (Wan *et al*. 2016) and *N_1_* was proposed to be one of the loci underlying the fiberless phenotype of ‘SL1-7-1’ (Turley and Kloth 2008). Loss-of-function of *MYB25-like_At* was thus considered to be linked to the fiberless trait of ‘SL1-7-1’.

None of the SS F_2_ segregants was completely fiberless and the lint% of the 79 fuzzless segregants ranged from 1.09 to 40.93%. Previous genetic analysis suggested that the fiberless trait of ‘SL1-7-1’ is controlled by 3 mutated loci (*N_1_N_1_n_3_n_3_fl_1_fl_1_*; Turley and Kloth 2008) although *n_3_* was recently proposed to be allelic to *N_1_* (Chen *et al*. 2020). ‘SL1-7-1’ was thus crossed to ‘T586’, containing *N_1_N_1_* or dysfunctional *MYB25-like_At*, to generate a segregating population (ST F_2_ with 101 plants) for identifying other genetic component(s) responsible for the fiberless trait of ‘SL1-7-1’ (Fig. 1). F_3_ seeds of all 101 ST F_2_ plants were fuzzless, providing supporting evidence for dysfunctional *MYB25-like_At* in ‘SL1-7-1’. Lint% of the F_2_ segregants ranged from 0 to 33.17% with 7 segregants having a lint% (0–1.46%) similar to that of ‘SL1-7-1’ (1.01%). Two DNA pools were prepared for BSA. The low lint% pool included 19 plants with a lint% < 5% (0–4.91%) and the high lint% pool included 19 plants with a lint% > 20% (20.13–33.17%). Reads were mapped to the in-house ‘T586’ genome sequence for SNP calling. Based on SNP frequency over the 26 cotton chromosomes, the best candidate region responsible for the low lint% was located to a ∼6.5-Mbp interval on Chr-A06 (Fig. 2b; Supplementary Fig. 3). The region was then narrowed down to ∼4.7 Mbp containing *HD1_At* based on the association of low lint% with the ‘SL1-7-1’ alleles that were genotyped using KASP markers (Supplementary Fig. 4). When the 101 F_2_ segregants (all with dysfunctional *MYB25-like_At*) were separated into 3 groups based on their *HD1_At* genotype determined by the KASP marker AM322 within *HD1_At* (Supplementary Fig. 5), the average lint% of *HD1_At^SL/SL^*, *HD1_At^SL/T586^*, and *HD1_At^T586/T586^* was 5.14, 13.75, and 20.47%, respectively. Most *HD1_At^SL/SL^* plants (16 out of 22) had a lint% < 5%, while most *HD1_At^T586/T586^* plants (16 out of 26) had a lint% > 20% (Table 1). These results indicate that ‘SL1-7-1’ contains a dysfunctional *HD1_At* allele although the causative mutation is yet to be identified.

**Table 1.** Distribution of the ST F_2_ (‘SL1-7-1’ × ‘T586’) plants that are homozygous ‘SL1-7-1’ (*HD1_At^SL/SL^*), heterozygous (*HD1_At^SL/T586^*), or homozygous ‘T586’ (*HD1_At^T586/T586^*) at the *HD1_At* locus in different ranges of lint percentage.

Genotype at *HD1-At*	0	0.01–5%	5.01–10%	10.01–15%	15.01–20%	20.01–25%	25.01–30%	>30%	Total no. of plants	Mean (%)^a^
*HD1_At^SL/SL^*	1	15	3	1	2	0	0	0	22	5.14 a
*HD1_At^SL/T586^*	0	5	12	10	21	3	2	0	53	13.75 b
*HD1_At^T586/T586^*	0	0	0	5	5	12	3	1	26	20.47 c

^a^Different letters indicate statistical significance at *P* < 0.05 based on 2-tailed Student's *t*-test.

**Fig. 3.**
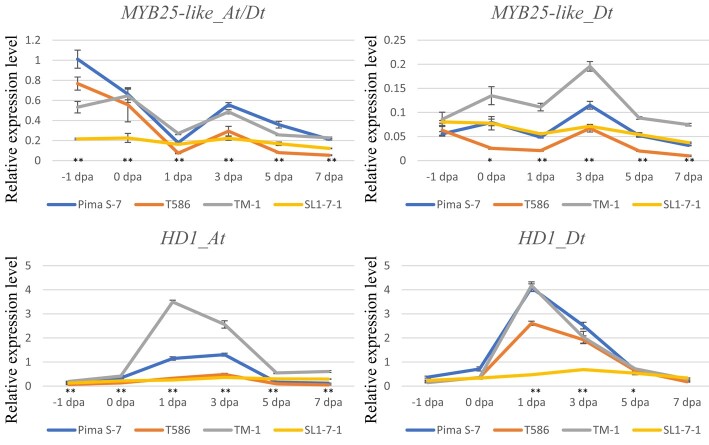
qRT-PCR-based expression profile of *MYB25-like* and *HD1* in −1 day-post-anthesis (dpa) to 7 dpa ovules of ‘Pima S-7’, ‘TM-1’, ‘T586’, and ‘SL1-7-1’. Error bars represent standard deviations. * and ** represent significance between ‘SL1-7-1’ and ‘TM-1’ at *P* < 0.05 and *P* < 0.01, respectively, based on 2-tailed Student's *t*-test.

In line with the genetic analysis results, ‘SL1-7-1’ had an expression level of *MYB25-like_At/Dt* in −1 to 0 dpa ovules (a critical period for fiber initiation) lower than ‘T586’ and an expression level of *HD1_At* in 0 to 5 dpa ovules lower than ‘Pima S-7’. ‘T586’ and ‘Pima S-7’ contain a dysfunctional *MYB25-like_At* and *HD1_At*, respectively (Ding *et al*. 2015; Wan *et al*. 2016; Niu *et al*. 2019). In addition, *HD1_Dt* was constantly very lowly expressed in −1 to 7 dpa ovules of ‘SL1-7-1’, implying *HD1_Dt* might be dysfunctional in ‘SL1-7-1’ as well (Fig. 3; Supplementary File 1).

### *HD1_At* of ‘Pima S-7’ contributes to low lint percentage

Our previous investigation indicated that, in addition to dysfunctional *MYB25-like_At* and *MYB25-like_Dt*, other yet to be identified dysfunctional genetic component(s) also contribute to the fiberless phenotype observed in the progeny derived from ‘T586’ × ‘Pima S-7’ (TP; Zhu *et al*. 2018).

To identify those additional component(s), a fiberless F_2_ progeny plant (TP F_2_-90) from ‘T586’ × ‘Pima S-7’ was backcrossed to ‘T586’ to generate the TPT BC_1_F_2_ population (Fig. 1). TP F_2_-90 had homozygous ‘T586’ alleles at both *MYB25-like_At* and *MYB25-like_Dt*, i.e. *MYB25-like_At^T586/T586^* and *MYB25-like_Dt^T586/T586^*, so the TPT BC_1_F_2_ population would not segregate for these 2 loci. As expected, seeds of all 118 TPT BC_1_F_2_ plants were fuzzless due to both parents containing *MYB25-like_At^T586/T586^*. Of the 118 progeny, 26 were fiberless and 92 had a lint% of 0.43–17.93%. Fifteen fiberless segregants and 15 segregants with a lint% > 11% (11.16–17.93%) were used in preparing the fiberless and high lint% DNA pool, respectively, for BSA. After mapping the reads to the in-house ‘T586’ genome sequence, we found that the region containing *HD1_At* (Chr-A06) was the best candidate region contributing to low lint% (Fig. 2c; Supplementary Fig. 6). The genomic interval with *HD1_At* was narrowed down to ∼6.0 Mbp based on association between lint% of the 118 progeny and their genotypes in the interval determined by KASP markers (Supplementary Fig. 7). When the 118 progeny were separated into 3 groups, i.e. *HD1_At^PS7/PS7^*, *HD1_At^PS7/T586^*, and *HD1_At^T586/T586^*, based on their *HD1_At* genotypes determined using the KASP marker AM294 within *HD1_At* (Supplementary Fig. 5), the average lint% of *HD1_At^PS7/PS7^* plants (0.24%) was significantly lower than that of other 2 groups and 26 of the 29 *HD1_At^PS7/PS7^* progeny were fiberless (Table 2), indicating that the *HD1_At* locus of ‘Pima S-7’ contributes to defective fiber initiation and low lint%.

**Table 2.** Distribution of the TPT BC_1_F_2_ [(‘T586’ × ‘Pima S-7’) × ‘T586’] segregants with homozygous ‘T586’ alleles at both *MYB25-like_At* and *MYB25-like_Dt* loci in different ranges of lint percentage.

Genotype at *HD1_At*	0	0.01–5%	5.01–10%	10.01–15%	15.01–20%	Total no. of plants	Mean (%)^a^
*HD1_At^PS7/PS7^*	26	3	0	0	0	29	0.24 a
*HD1_At^PS7/T586^*	2	27	24	4	0	57	5.17 b
*HD1_At^T586/T586^*	0	4	11	12	5	32	10.43 c

^a^Different letters indicate statistical significance at *P* < 0.05 based on 2-tailed Student's *t*-test.

### Association of *HD1_Dt* with lint percentage

TP F_2_-90 (with homozygous *MYB25-like_At^T586/T586^* that is dysfunctional according to Wan *et al*. (2016)) was also backcrossed to ‘Pima S-7’ (with dysfunctional *MYB25-like_Dt* and *HD1_At* according to our previous results (Zhu *et al*. 2018) and the results presented above) to generate the TPP BC_1_F_2_ population with 126 segregants. Although the fuzzless trait of the population fitted the segregation ratio (95 fuzzless vs 31 fuzzy, *X*^2^ = 0.011, *P* = 0.9181) of a single dominant mutation (due to the presence of *MYB25-like_At^T586/T586^*), only 1 segregant (out of 126) showed a fiberless seed phenotype, implying that the fiberless seed trait is conferred by more than 3 genetic components. This was supported by the observation that, among the 126 segregants, several had identical dysfunctional *MYB25-like_At*, *MYB25-like_Dt*, and *HD1_At* alleles, i.e. *MYB25-like_At^T586/T586^*, *MYB25-like_Dt^PS7/PS7^*, and *HD1_At^PS7/PS7^*, but produced seeds from fiberless to bearing a reasonable amount of lint. Hence, 2 of such segregants (TPP BC_1_F_2_-51: fiberless; TPP BC_1_F_2_-137: fuzzless and with the highest lint%—10.10%) were crossed to generate a secondary segregating population (TPP SBC_1_F_2_) to identify the fourth locus responsible for initiation of lint fiber.

The TPP SBC_1_F_2_ population included 106 plants with 20 and 86 being fiberless and linted (lint%: 0.26–13.19%), respectively. Of the 86 linted segregants, only 5 had a lint% similar to or greater than the linted parent (TPP BC_1_F_2_-137, 10.10%; Table 3). BSA was conducted using the 20 fiberless segregants and 11 segregants with a lint% > 7% (7.75–13.19%). According to the read mapping and SNP call results based on the in-house ‘T586’ genome sequence, the Chr-D06 region containing *HD1_Dt* was identified to be the best candidate (Fig. 2d; Supplementary Figs. 8 and 9). A KASP marker (AM348, Supplementary Fig. 5) diagnostic for the *HD1_Dt* allele of ‘T586’ and ‘Pima S-7’ was used to genotype all 106 plants to separate them into 3 groups, i.e. *HD1_Dt^T586/T586^*, *HD1_Dt^T586/PS7^*, and *HD1_Dt^PS7/PS7^*. The average lint% of these 3 types of plants (all with the same dysfunctional allele at *MYB25-like_At*, *MYB25-like_Dt*, and *HD1_At*) was 0.41%, 3.39%, and 5.79%, respectively. The plants with the homozygous *HD1_Dt* allele of ‘T586’ tended to be fiberless or had lower lint% (Table 3), suggesting a negative effect of *HD1_Dt^T586/T586^* on lint initiation.

**Table 3.** Distribution of the TPP^a^ SBC_1_F_2_ segregants with the genetic background of *MYB25-like_At^T586/T586^*, *MYB25-like_Dt^PS7/PS7^*, and *HD1_At^PS7/PS7^* in different ranges of lint percentage.

Genotype at *HD1_Dt*	0	0.01–1%	1.01–3%	3.01–5%	5.01–10%	10.01–15%	Total no. of plants	Mean (%)^b^
*HD1_Dt^T586/T586^*	16	6	6	0	0	0	28	0.41 a
*HD1_Dt^T586/PS7^*	4	6	22	12	13	1	58	3.39 b
*HD1_Dt^PS7/PS7^*	0	2	5	4	5	4	20	5.79 c

^a^TPP SBC_1_F_2_: [(‘T586’ × ‘Pima S-7’) × ‘Pima S-7’] (fiberless) × [(‘T586’ × ‘Pima S-7’) × ‘Pima S-7’] (lint%: 10.10%).

^b^Different letters indicate statistical significance at *P* < 0.05 based on 2-tailed Student's *t*-test.

### *HD1_At* has a larger effect on lint percentage than *HD1_Dt*

We first evaluated the impact of *HD1_At* of ‘T586’ and ‘Pima S-7’ on lint development in 2 F_3_ populations derived from TP F_2_-22 and TP F_2_-55 and segregating for *HD1_At*. While the lint% of the TP F_2_-22 progeny was much lower than that of the TP F_2_-55 progeny (Table 4), thanks to dysfunctional *MYB25-like_At* of TP F_2_-22 (Supplementary Table 2), the average lint% of the 3 types of segregants with different *HD1_At* allele was *HD1_At^T586/T586^* > *HD1_At^T586/PS7^* > *HD1_At^PS7/PS7^* in both populations (Table 4), indicating the negative effect of *HD1_At^PS7/PS7^* on lint% being stronger than that of *HD1_At^T586/T586^* in different genetic background (Supplementary Table 2).

**Table 4.** The effect of the *HD1_At* and *HD1_Dt* loci on lint percentage.

Genotype at *HD1_At*	TP F_2_-22	TP F_2_-55	Genotype at *HD1_At*	Genotype at *HD1_Dt*	TP F_2_-65	TP F_2_-88
	Lint%^a^	Lint%			Lint%	Lint%
*HD1_At^T586/T586^*	10.44 a	35.42 a	*HD1_At^T586/T586^*	*HD1_Dt^T586/T586^*	15.75 a	33.19 a
*HD1_At^T586/PS7^*	3.51 b	30.58 b		*HD1_Dt^T586/PS7^*	16.86 a	34.39 a
*HD1_At^PS7/PS7^*	0.92 c	26.04 c		*HD1_Dt^PS7/PS7^*	16.41 a	n/a
			*HD1_At^T586/PS7^*	*HD1_Dt^T586/T586^*	12.15 b	31.05 ab
				*HD1_Dt^T586/PS7^*	12.88 b	28.44 b
				*HD1_Dt^PS7/PS7^*	15.02 a	29.99 ab
			*HD1_At^PS7/PS7^*	*HD1_Dt^T586/T586^*	2.05 d	27.41 b
				*HD1_Dt^T586/PS7^*	7.57 c	27.69 b
				*HD1_Dt^PS7/PS7^*	7.76 c	27.42 b

^a^Different letters indicate statistical significance at *P* < 0.05 based on 2-tailed Student's *t*-test.

We then assessed the relative effect of *HD1_At* and *HD1_Dt* on lint development using 2 F_3_ populations derived from TP F_2_-65 and TP F_2_-88 and segregating for both loci. TP F_2_-65 and TP F_2_-88 had the same genotype at *MYB25-like_Dt* (*MYB25-like_Dt^T586/T586^*) but differing at *MYB25-like_At* (Supplementary Table 2). Because of the dysfunctional *MYB25-like_At* in TP F_2_-65, the overall lint% of the TP F_2_-65 population was much lower than that of the TP F_2_-88 population. As in the F_3_ populations from TP F_2_-22 and TP F_2_-55 mentioned above, the negative impact of the ‘Pima S-7’ *HD1_At* allele on lint development was stronger than that of the ‘T586’ *HD1_At* allele, particularly in TP F_2_-65 (Table 4). In the TP F_2_-65 population, when *HD1_At* was homozygous for the ‘T586’ allele, the *HD1_Dt* allele, whether it was from ‘T586’ or ‘Pima S-7’, had little effect on lint%; however, when *HD1_At* was heterozygous (*HD1_At^T586/PS7^*), the plants with homozygous ‘Pima S-7’ *HD1_Dt* (i.e. *HD1_Dt^PS7/PS7^*) had a significantly higher lint% than the plants with *HD1_Dt^T586/T586^* or *HD1_Dt^T586/PS7^*; when *HD1_At* was homozygous for the ‘Pima S-7’ allele (i.e. *HD1_At^PS7/PS7^*), both *HD1_Dt^PS7/PS7^* and *HD1_Dt^T586/PS7^* plants had a significantly higher lint% than the *HD1_Dt^T586/T586^* plants (Table 4). These results confirmed the negative impact of the ‘T586’ *HD1_Dt* allele on lint development and suggest that its impact is genetic background dependent. In the TP F_2_-88 population, thanks to the presence of a functional *MYB25-like* (i.e. *MYB25-like_At^PS7/PS7^* and *MYB25-like_Dt^T586/T586^*), the impact of the *HD1_Dt* allele of ‘T586’ and ‘Pima S-7’ on lint development was hardly distinguishable, although the lint% of the plants with *HD1_At^PS7/PS7^* tended to be lower than that of the plants with *HD1_At^T586/T586^* or *HD1_At^T586/PS7^* (Table 4). Together, these results indicate that *HD1_At* has a larger impact on lint development than *HD1_Dt*.

### *MYB25-like_At* and *HD1_At* are the major loci responsible for lint development

To further uncouple the relative effects of the individual locus of *MYB25-like* and *HD1* on lint development and lint%, the 303 TP F_2_ plants (Fig. 1) were genotyped using diagnostic KASP markers for the 4 genetic loci (Supplementary Fig. 5) and measured for their lint%. The average lint% of plants with homozygous dysfunctional alleles of *MYB25-like_At*, *MYB25-like_Dt*, *HD1_At*, or *HD1_Dt* were 12.08%, 19.10%, 16.83%, and 20.02%, respectively (Table 5), suggesting that, when mutated, their negative effect on lint% are *MYB25-like_At* > *HD1_At* > *MYB25-like_Dt* > *HD1_Dt*. For *MYB25-like_At*, the lint% was significantly different among the plants with a genotype of *MYB25-like_At^T586/T586^* (i.e. homozygous ‘T586’ alleles), *MYB25-like_At^T586/PS7^* (heterozygous), or *MYB25-like_At^PS7/PS7^* (homozygous ‘Pima S-7’ alleles). For *HD1_At*, although the lint% of the *HD1_At^T586/PS7^* plants was only insignificantly lower than that of *HD1_At^T586/T586^*, the lint% of the *HD1_At^PS7/PS7^* plants was significantly lower than that of *HD1_At^T586/T586^* and *HD1_At^T586/PS7^*. For *MYB25-like_Dt*, the lint% of the *MYB25-like_Dt^PS7/PS7^* plants was significantly lower than that of *MYB25-like_Dt^T586/T586^*, but the lint% of the heterozygote (*MYB25-like_Dt^T586/PS7^*) was insignificantly different from that of either homozygote. For *HD1_Dt*, while the ‘T586’ allele had a negative effect on lint%, the effect was not significant (Table 5). Of the 303 TP F_2_ progeny, only 3 that had homozygous dysfunctional alleles in all 4 loci or in 3 loci with the fourth being heterozygous for *MYB25-like_Dt^T586/PS7^* produced completely fiberless seeds. All TP F_2_ progeny with homozygous loss-of-function alleles of *MYB25-like_At* (*MYB25-like_At^T586/T586^*) and *HD1_At* (*HD1_At^PS7/PS7^*) had a lint% < 10%, regardless the genotypes at other 2 loci (Supplementary Table 3). These results reassert the importance of *MYB25-like_At* and *HD1_At* in lint initiation and development.

**Table 5.** The effect of the individual *MYB25-like* and *HD1* locus on lint percentage.

Genotype	*MYB25-like_At*	*MYB25-like_Dt*	*HD1_At*	*HD1_Dt*
Homozygous T586	12.08 a^a^	21.78 a	23.22 a	20.02 a
Heterozygous	22.19 b	20.44 ab	21.14 a	20.63 a
Homozygous Pima S-7	25.33 c	19.10 b	16.83 b	20.69 a

^a^Different letters indicate statistical significance at *P* < 0.05 based on 2-tailed Student's *t*-test.

### Haplotypes of *MYB25-like* and *HD1* and their association with lint percentage in a *G. hirsutum* diversity panel

**Fig. 4.**
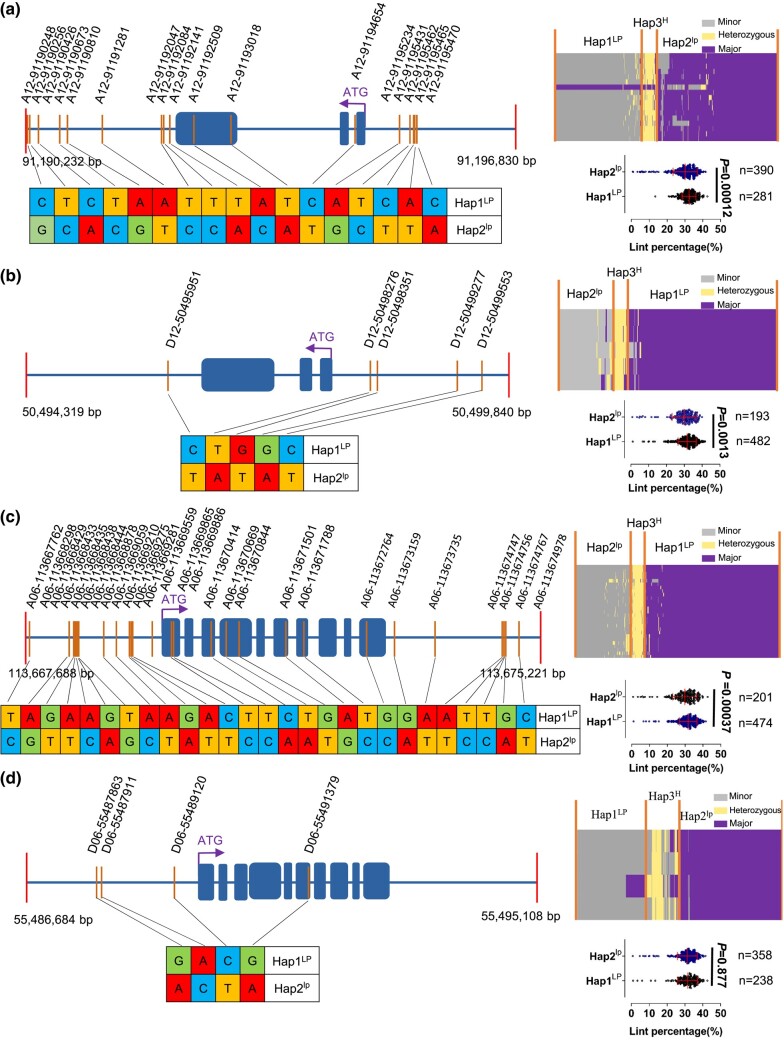
Haplotypes of the *MYB25-like_At*, *MYB25-like_Dt*, *HD1_At*, and *HD1_Dt* loci (including 2-kb flanking region) and their association with lint percentage in the 723 *G. hirsutum* accessions. a) *MYB25-like_At*. b) *MYB25-like_Dt*. c) *HD1_At*. d) *HD1_Dt*. At each locus, the left panel shows the SNPs in the annotated gene with the haplotype of LP (higher lint percentage) and lp (lower lint percentage) shown below. Dark blue rectangles represent exons. The purple arrow indicates transcription orientation of the gene. On the right, the top is a haplotype heatmap with the *X*-axis representing individual accessions grouped based on the SNPs of the locus distributing across the *Y*-axis; the dot plot shows the association of the LP and lp haplotypes with lint percentage (each dot representing an accession). In each dot plot, the left and right vertical red lines represent quartiles and the middle one median. The significance of association between lint percentage and the LP or lp haplotype is indicated by the *P*-value (based on Mann–Whitney test).

Next, we used a *G. hirsutum* diversity panel to investigate the effect of *MYB25-like* and *HD1* on lint%. SNP-based haplotype analysis separated the 723 *G. hirsutum* accessions (including 81 fuzzless seed accessions; Supplementary Table 4) into 3 haplotypes (LP and lp for higher and lower lint%, respectively, and heterozygous) at each of the *MYB25-like* and *HD1* loci. At the *MYB25-like_At* (*P* = 1.2 × 10^−4^), *MYB25-like_Dt* (*P* = 1.3 × 10^−3^), and *HD1_At* (*P* = 3.7 × 10^−4^) loci, the LP accessions had a significantly higher lint% than the lp accessions (Supplementary Figs. 4a, 4b, and 4c), but at the *HD1_Dt* locus (*P* = 0.877), the lint% difference between the LP and lp accessions was insignificant (Fig. 4d).

Regarding individual SNPs, we found a nonsynonymous SNP (A vs T at A12-91193018) located in the 3rd exon of *MYB25-like_At* being significantly associated with lint% (*P* = 2.72 × 10^−25^), with the TT allele being the LP allele and present in most (84.76%) of the *G. hirsutum* accessions, particularly among the fuzzy seeded accessions (Fig. 4a; Table 6). The SNP converts the 105th amino acid (within the 2nd MYB-DNA binding domain) of *MYB25-like_At* from lysine (AAG) to methionine (AUG), which may affect the DNA binding ability of MYB25-like_At and hence its function.

**Table 6.** The mean lint percentage (%) of the 3 haplotypes of each gene in accessions with fuzzless or fuzzy seeds.

Gene	Fuzzless seed accessions			Fuzzy seed accessions		
	lp	Hetero	LP	lp	Hetero	LP
*MYB25-like_At* ^a^	19.45 (7.98)^b^	24.77 (2.42)	26.58 (1.00)	29.88 (2.14)	31.12 (2.71)	32.33 (83.76)
*MYB25-like_At*	20.96 (10.25)	22.75 (0.42)	27.63 (0.42)	32.15 (43.91)	32.37 (6.51)	32.35 (38.50)
*MYB25-like_Dt*	18.49 (3.88)	20.14 (0.97)	23.20 (6.23)	31.60 (22.99)	33.22 (5.26)	32.42 (60.66)
*HD1_At*	19.57 (4.84)	16.40 (1.11)	23.78 (5.26)	31.73 (22.96)	33.34 (5.39)	32.36 (60.44)
*HD1_Dt*	18.87 (3.46)	23.07 (4.98)	20.98 (2.77)	32.23 (46.20)	32.02 (12.45)	32.51 (30.15)

^a^Based on the SNP in the 3rd exon of *MYB25-like_At*. LP and lp refer to the TT and AA genotype, respectively.

^b^The number in parentheses represents the proportion of accessions with the corresponding haplotype.

When the 723 *G. hirsutum* accessions were separated into fuzzless (81) and fuzzy (642) groups, for each of the 4 genes; first, the average lint% of the fuzzy seed accessions was higher than that of the fuzzless accessions regardless of their haplotypes (Table 6), suggesting a negative impact of the fuzzless gene(s) on lint%, likely due to a pleiotropic effect of the fuzzless gene(s) on lint development; second, the difference of the average lint% between the LP and lp haplotype in the fuzzless group was much larger than that in the fuzzy group (Table 6), implying that the negative impact of the fuzzless gene(s) on lint% can be partially compensated for by the presence of the LP haplotype of each gene. Among the fuzzy seeded accessions, the number (and proportion) of the accessions with the LP haplotype was more (and higher) than that with the lp allele/haplotype at the *MYB25-like_Dt* and *HD1_At* loci, but less (and lower) at the *MYB25-like_At* and *HD1_Dt* loci. However, at the *MYB25-like_At* locus, when the accessions were grouped based on the A12-91193018 SNP, most fuzzy seeded accessions but not fuzzless seed accessions had the LP allele (TT) (Table 6). Among the fuzzless accessions, at the *MYB25-like_At* locus, there were many more lp accessions than LP ones; while at the *HD1_Dt* locus, the number of lp accessions was only slightly more than that of LP accessions; in contrast, slightly more LP accessions than lp accessions were observed at the *MYB25-like_Dt* locus; and an equal number of LP and lp accessions were observed at the *HD1_At* locus (Table 6). These results also showed a strong correlation between the fuzzless seed trait and low lint% at the *MYB25-like_At* locus and a decreasing correlation in the order of *MYB25-like_Dt*, *HD1_At*, and *HD1_Dt*, in line with *MYB25-like* being important for the development of both fuzz and lint fibers (Zhu *et al*. 2018). Nevertheless, the lp allele (AA) at A12-91193018 itself did not seem to contribute to the fuzzless seed trait because ∼2% of the fuzzy accessions contained this allele (Table 6).

The same approach was applied to a *G. barbadense* population with 326 accessions (Supplementary Table 5) for identification of haplotypes at the 4 genetic loci. At the *HD1_At* locus, the 326 accessions could be grouped into 3 haplotypes (LP, hetero, and lp), with majority of the accessions (73.0%) containing the lp haplotype; however, the average lint% of the 3 haplotypes was not significantly different (Supplementary Fig. 10). Given that the loss-of-function of *HD1_At* in *G. barbadense* caused by a retrotransposon insertion is associated with hairless stems (Ding *et al*. 2015; Niu *et al*. 2019), it will be of interest to know the association between the haplotypes and the density of their stem trichomes. Only a single SNP and a few SNPs without linkage relationships were found at the *MYB25-like_At* and *HD1_Dt* loci, respectively, so no haplotype could be defined. At the *MYB25-like_Dt* locus, no SNP was found among the 326 accessions. These results indicate that the *G. barbadense* population used here has a relatively uniform genetic make-up in 3 of the 4 genetic loci and imply that these loci may have been fixed by natural and artificial selection because of their importance in the determination of lint development.

### The effect of allelic combinations on lint percentage

**Fig. 5.**
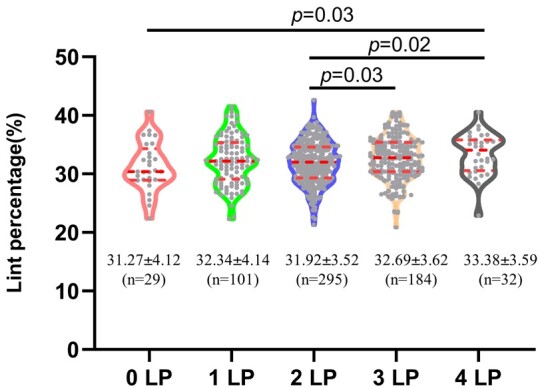
Violin plot shows the effect of the number of LP alleles at the 4 loci on lint percentage of the fuzzy seeded *G. hirsutum* accessions. The mean lint percentage and the number of accessions of each group are shown below the violin image, with those being significantly different (*P* < 0.05; based on Mann–Whitney test) indicated by the *P*-value. The dot lines represent quartiles (the top and bottom ones) and medians (the middle ones). Each gray dot represents an accession.

Given that nearly all commercial Upland cotton varieties have the fuzzy seed trait, we further explored the effect of the favorable haplotype (i.e. the LP allele) of the 4 loci on lint% using the fuzzy seeded *G. hirsutum* accessions (Supplementary Table 4). Of the 641 accessions that could be used in the analysis, 29 (4.52%) lacked the LP allele (0 LP) at all 4 loci, and 15.76%, 46.02%, 28.71%, and 4.99% of the accessions carried 1, 2, 3, and 4 LP alleles, respectively. The proportion of the accessions with 0, 1, and 4 LP alleles was lower than the expected (6.25%, 25%, and 6.25%, respectively), while that with 2 and 3 LP alleles was higher than the expected (37.5 and 25%, respectively), indicating that the LP alleles of the 4 loci, with different combinations of 2 or 3 but not all 4, have been preferentially retained through breeding practices. The mean lint% of the accessions with 4, 3, 2, 1, and 0 LP allele were 33.38%, 32.69%, 31.92%, 32.34%, and 31.27%, respectively. The mean lint% of the accessions with 3–4 LP alleles was significantly higher than that with 2 LP alleles (Fig. 5), indicating that combining more LP alleles at the *MYB25-like* and *HD1* loci tends to increase lint%. Similarly, the pyramiding effect of LP alleles on lint% was reported for 2 other candidate genes associated with lint% (Chen *et al*. 2022).

Nevertheless, we also noticed significant variation of lint% within each group that contained 1–4 LP alleles, and a high lint% was observed even in some fuzzless seed accessions, such as sGuokang36 (41.6%; Supplementary Table 4), implying that, while *MYB25-like* and *HD1* are the major genes in the determination of lint%, development of lint fibers is regulated by complicated gene networks, which involve many other genetic loci that act independently or interactively with *MYB25-like* and *HD1*.

## Discussion

Improving lint yield is the final goal of every cotton breeding program. Cotton lint yield is determined by seed cotton yield and lint%. Three strategies can be adopted to achieve the goal of high lint yield—enhancing seed cotton yield without changing lint%, increasing lint% without changing seed cotton output, and improving both seed cotton yield and lint%. Seed cotton yield is often affected by environmental conditions, while lint% is mainly genetically determined and less affected by environmental conditions compared to seed cotton yield (Zhu, Hou, *et al*. 2021). Increasing lint% has thus been the main strategy adopted by cotton breeders for improving overall cotton lint yield. As a result, lint% of modern commercial cotton varieties is much higher than those of the older obsolete varieties (Ma *et al*. 2019; Conaty and Constable 2020), however this comes with a tradeoff in the size and nutritional composition of the cotton seeds (that tend to get smaller), causing negative effect on seed germination, seedling vigor, and on the downstream industries using cotton seeds as raw materials (Maeda *et al*. 2023).

Given the importance of lint% in developing high-yielding elite commercial cotton varieties, numerous studies, including genetic mapping using segregating populations derived from biparental crosses and GWAS based on natural populations or germplasm collections, have identified hundreds of lint% QTLs (Niu *et al*. 2022). Individually, most lint% QTLs have a small genetic effect and are population dependent, so are rarely applicable in different breeding programs. The GWAS-based QTLs may be more breeder-friendly than the QTLs identified based on specific biparental crosses, but they may also be only relevant to the accessions used in the analysis, making them less likely to be useful in breeding programs based on different germplasm. Identifying major genetic loci responsible for lint% and knowing how they were shaped to regulate lint% during cotton evolution and by historical breeding practices will provide practical genetic solutions for improving lint%, and consequently lint yield of cotton, on the basis of without compromising the development of cotton seeds.

Fuzzless and fiberless mutants provide a unique opportunity to study the genetic mechanisms underlying lint initiation and to shed insight on enhancing lint development and improving lint%, because the number of lint fiber initials is one of the major determinants of lint%. The fuzzless seed phenotype of the dominant *N_1_* mutant is because of the loss-of-function of *MYB25-like_At* (Wan *et al*. 2016), and that of the recessive *n_2_* mutant is likely to be caused by a dysfunctional *MYB25-like_Dt* (Zhu *et al*. 2018). Significant variation in lint% (0.7–23.6%) has been observed in different fuzzless seed accessions (Turley *et al*. 2007), and fiberless segregants were found among the progeny derived from crosses between the *N_1_* and *n_2_* mutants (Turley and Kloth 2002; Zhu *et al*. 2018; this study), suggesting that *MYB25-like_At* alone, *MYB25-like_Dt* alone, and interactions between these 2 homeologs, or their interaction with other gene(s) play an important role in the determination of initiation of lint fibers and lint%. In this study, the fiberless seed phenotype of ‘SL1-7-1’ was linked to dysfunctional *MYB25-like_At* and *HD1_At* (Figs. 2, a and b, and 3). Further investigation using a population derived from the cross between lines with both *MYB25-like_At* and *MYB25-like_Dt* mutated but with different lint% also linked the *HD1_At* locus to initiation of lint fibers and lint% (Fig. 2c). Using a population derived from the cross between lines with dysfunctional *MYB25-like_At*, *MYB25-like_Dt*, and *HD1_At*, but still showing different lint% further identified the *HD1_Dt* locus to be another genetic component associated with initiation of lint fiber and lint% (Fig. 2d). Comprehensive analyses of the genetic effect of single and different combinations of the 4 loci in different genetic backgrounds and the *G. hirsutum* diversity panel revealed that while the negative impact of dysfunctional *MYB25-like_Dt*, *HD1_At*, and *HD1_Dt* on lint% is often masked by the presence of a functional *MYB25-like_At*, it is clear that both homeologs of *MYB25-like* and *HD1*, especially *MYB25-like_At* and *HD1_At*, are the major genes determining initiation of lint fiber and consequently lint%. In line with this observation, at the key time points of fiber initiation (from −1 to 0 dpa), *MYB25-like_At* and *HD1_At* are the 2 most important transcription factors (TFs) at −1 dpa, and *HD1_At* and *MYB25-like_Dt* are the 2 most important TFs at −0.5 dpa involved in regulating fiber initiation. At 0 dpa, while *MYB25-like_Dt* and *HD1_At* still play a key role in regulating the gene networks underlying fiber initiation, *HD1_Dt* also becomes one of the key TFs of the networks (Qin *et al*. 2022).

*MYB25-like* genes also regulate initiation and development of fuzz fibers (Wan *et al*. 2016; Zhu *et al*. 2018). According to the SNP-based genotype information, 723 *G. hirsutum* accessions could be separated into 3 alleles (LP, hetero, and lp) at *MYB25-like_At* and *MYB25-like_Dt* (Figs. 4a and 4b). Both LP and lp alleles were found in accessions with fuzzy or fuzzless seeds, suggesting that the lp alleles themselves are not the causes of the fuzzless seed phenotype. In other words, the sequence variants of the lp haplotype are only associated with development of lint fibers but not fuzz fibers. Among the fuzzy seeded accessions, more contain the LP allele at *MYB25-like_Dt* and *HD1_At*, and at *MYB25-like_At*, the LP allele at A12-91193018, a SNP identified to be associated with higher lint%, was the predominant one (Table 6); meanwhile the lint% of the accessions containing 3–4 LP alleles was significantly higher than those with 2 LP alleles (Fig. 5). These observations suggest that the LP alleles of *MYB25-like* and *HD1* have experienced positive selection in breeding practices though further testing is required for confirmation, and that pyramiding the LP alleles of the 4 loci would be the basis for further improving lint%.

None of previous QTL mapping and GWAS studies had identified *MYB25-like* or *HD1* as being associated with lint% (Niu *et al*. 2022). One reason could be that these loci have been almost completely fixed due to their importance in determining initiation of lint fiber and lint%, as indicated by the observation that most fuzzy seeded *G. hirsutum* accessions contained the LP alleles/haplotypes at the 4 loci (Table 6, Supplementary Table 4) and no different haplotypes could be identified at 3 of the 4 loci in the *G. barbadense* accessions analyzed. Alternatively, the functionality of some loci may be repressed because of epistasis. In this study, a SNP in *MYB25-like*_*At* was found to be significantly associated with lint% thanks to the inclusion of fuzzless/fiberless accessions, with 68% of them containing the lp allele (11% with LP and 21% being heterozygous) and 33% of them having a lint% ≤ 20% (the lowest lint% of the fuzzy seeded accessions is 20.9%; Supplementary Table 4). Therefore, diversifying the genetic background and phenotype of the materials used is crucial for GWAS. These 2 factors also determine whether the genetic loci with relatively weak impact can be uncovered, particularly for those with epistatic relationships. For instance, we demonstrated *HD1_Dt* as one of the major loci in the determination of lint%, largely due to using of an innovative strategy in creating the segregating population, i.e. the 2 parents used had dysfunctional alleles in all known major loci affecting lint% but still showed distinct phenotypes. This strategy is generally applicable for dissecting the role of genes with redundant and/or additive functionality.

Given the observation of *MYB25-like* and *HD1* being the major genetic elements responsible for lint%, the straightforward approach for improving lint% seems to be to enhance their expression levels. Indeed, overexpressing *HD1_At* increases the number of fiber initials (Walford *et al*. 2012). But field experiments using transgenic cotton overexpressing *HD1_At* and *MYB25-like* individually or in combination showed that, generally, enhancing the expression of the gene(s) had little effect on lint% and lint yield, despite occasional observation of a few lines with higher lint% (Liu *et al*. 2020). One reason could be that while overexpressing *HD1_At* increases the number of fiber initials, they might not fully develop into lint fibers since increased numbers of short fibers were observed on the ginned seeds from the transgenic cotton overexpressing *HD1_At* (Walford *et al*. 2012). Simply enhancing the overall expression levels of *MYB25-like* and *HD1*, even when using an ovule-specific promoter (*FBP-7*), was unable to achieve what had been expected (Liu *et al*. 2020), implying that the expression levels of the genes involved in the biological processes following fiber initiation have to be tempo-spatially synchronized and coordinated with the increased expression of *MYB25-like* and *HD1* to ensure proper development of all fiber initials into lint fibers. Many genes have been individually demonstrated to be important for fiber elongation, cellulose synthesis, and secondary cell deposition, such as homeobox-leucine zipper gene *GhHOX3* (Shan *et al*. 2014), alanine-rich-protein gene *GhAlaRP* (Zhu, Li, *et al*. 2021), *GhTCP4* (TCP for *T*eosinte branched 1, *C*ycloidea, *P*CF1; Cao *et al*. 2020), and sucrose synthase gene *GhSusA1* (Jiang *et al*. 2012), but how they are regulating fiber development coordinately with *MYB25-like* and *HD1* and others, such as phytohormone related genes, is yet to be explored using systems biology approaches.

Given that the number of the LP alleles of *MYB25-like* and *HD1* seems to be positively correlated with lint% (Fig. 5), from a breeder's perspective, pyramiding the LP alleles of *MYB25-like* and *HD1* would be the first choice for improving lint%. Indeed, significant room still exists in terms of pyramiding the LP alleles of the 4 loci. For instance, among the fuzzy seeded *G. hirsutum* accessions used in this study, at the *MYB25-like_At* locus, while the majority already have the LP allele at A12-91193018, about half of the accessions still have the lp haplotype; at the other 3 loci, a quarter to half of the accessions still possess the lp allele (Table 6); and the proportion of the *G. hirsutum* accessions containing 4 LP alleles (4.99%) is slightly lower than the expected 6.25% (Fig. 5; Supplementary Table 4). The next step would be to check for the presence of the reported lint% QTLs using molecular markers and to investigate new lint% QTLs in the breeder's germplasm based on genetic mapping and/or GWAS. The known (if present) and the newly identified QTLs could then be further pyramided with the LP alleles of *MYB25-like* and *HD1*. Nevertheless, choosing the QTLs to be used in further pyramiding demands careful consideration so as to minimize or avoid any possible negative impact on other traits because almost all lint% QTLs characterized so far have pleiotropic negative effects on traits like seed size and fiber quality (Fang *et al*. 2017; Ma *et al*. 2019; Li *et al*. 2023). A first step will be to identify lint% QTLs without negative effects on other traits by evaluating all of those reported to date or by using new germplasm and populations to identify new ones. Because many of the reported lint% QTLs have pleiotropic negative effects, the lint% QTLs that contain genes, such as *Gh_D02G0025* (Ma *et al*. 2018) and *Gh_D07G0463* and *Gh_D01G0162* (Chen *et al*. 2022) with a potential role in enhancing fiber initiation and rapid elongation, might be an initial choice for pyramiding because they are less likely to have negative impact on seed development and fiber quality.

Loss-of-function of *MYB25-like* contributes to low lint% (Turley *et al*. 2007; Walford *et al*. 2011; Wan *et al*. 2016; Zhu *et al*. 2018), but the nonsynonymous A to T change in the 2nd MYB-DNA binding domain of *MYB25-like_At* seems to play a positive role in increasing lint% (Table 6). Incorporating the favorable TT allele in the background of the dominant fuzzless mutation *N_5_* (containing the AA allele of *MYB25-like_At*) that was reported to have a weaker impact on lint% (Zhu, Stiller, *et al*. 2021) would combine the fuzzless seed trait with high lint% to mitigate the negative impact of high lint% on seed development because the resources normally devoted to fuzz development would presumably be redirected for seed and/or lint development. The cutting-edge base editing technology would be the ideal approach for achieving this goal without extensive breeding (Qin *et al*. 2020), although the bottleneck of genotype-dependent transformation in cotton needs to be solved.

In summary, this study uncovered the homeologs of both *MYB25-like* and *HD1* as the major genetic components responsible for fiber initiation and lint% using fiber mutants and innovative methodology in the generation of segregating populations for dissecting the components in a step-by-step manner. The importance of *MYB25-like* and *HD1* in the determination of lint% was demonstrated using an Upland cotton population, in which the lint% of the accessions with ≥1 LP allele at *MYB25-like* and *HD1* was higher than those without any LP allele, especially those containing >2 LP alleles. Strategies for breeding high-yielding cotton varieties by improving lint% without compromising other important agronomic traits are proposed.

## Supplementary Material

iyad219_Supplementary_Data

## Data Availability

The raw sequencing data used in BSA are available at the CSIRO Data Access Portal by following the link: https://doi.org/10.25919/p728-ye68. The information about the fuzzy seeded *G. hirsutum* accessions and *G. barbadense* accessions can be found in PRJNA605345 and PRJNA637990, respectively. The lint% data of all populations used in the study are available in the text or in supplementary files that are available at GENETICS online. Supplemental material available at GENETICS online.
