# Supplementary material for: Dissecting the major genetic components underlying cotton lint development
Source: Genetics. 2023 Dec 26;226(2):iyad219. doi: 10.1093/genetics/iyad219 (PMC10847716; doi:10.1093/genetics/iyad219)
Supplement: iyad219_Supplementary_Data [file iyad219_supplementary_data.zip › Supplemental_Figures_GENETICS-2023-306698.pptx]

## Slide 1
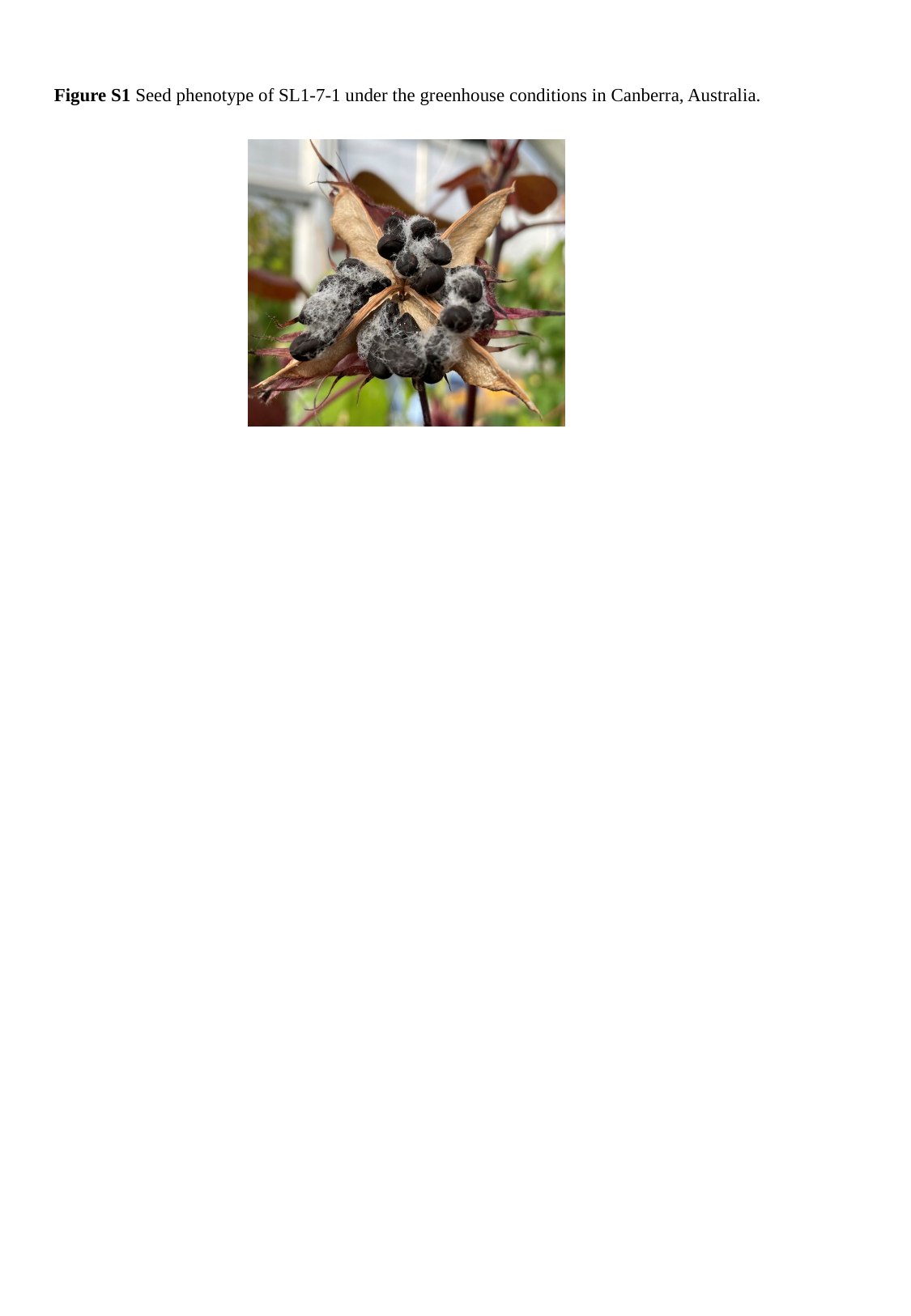

Figure S1 Seed phenotype of SL1-7-1 under the greenhouse conditions in Canberra, Australia.

## Slide 2
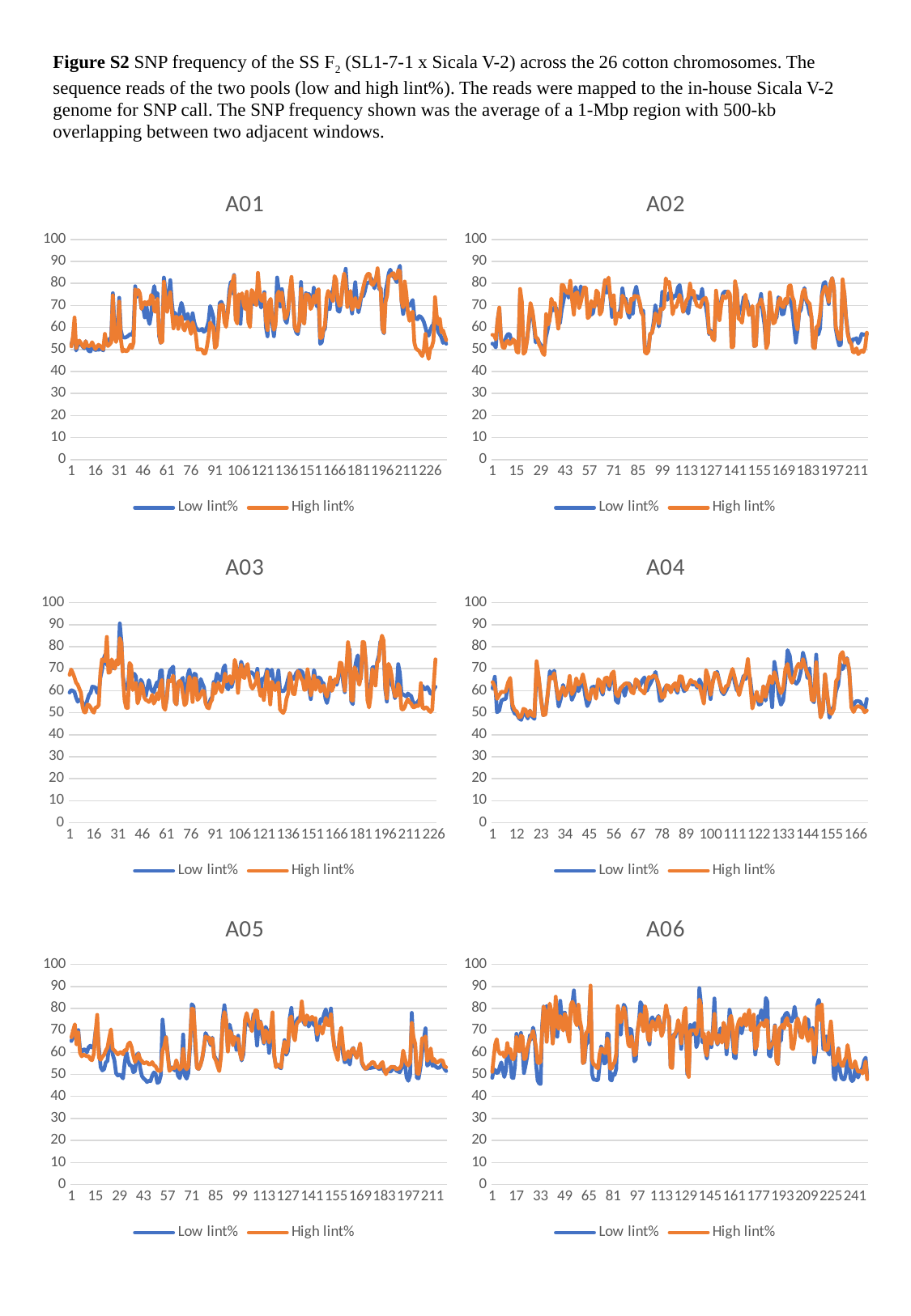

Figure S2 SNP frequency of the SS F2 (SL1-7-1 x Sicala V-2) across the 26 cotton chromosomes. The sequence reads of the two pools (low and high lint%). The reads were mapped to the in-house Sicala V-2 genome for SNP call. The SNP frequency shown was the average of a 1-Mbp region with 500-kb overlapping between two adjacent windows.
### Chart: A01
| Category | Low lint% | High lint% |
|---|---|---|
### Chart: A02
| Category | Low lint% | High lint% |
|---|---|---|
### Chart: A03
| Category | Low lint% | High lint% |
|---|---|---|
### Chart: A04
| Category | Low lint% | High lint% |
|---|---|---|
### Chart: A05
| Category | Low lint% | High lint% |
|---|---|---|
### Chart: A06
| Category | Low lint% | High lint% |
|---|---|---|

## Slide 3
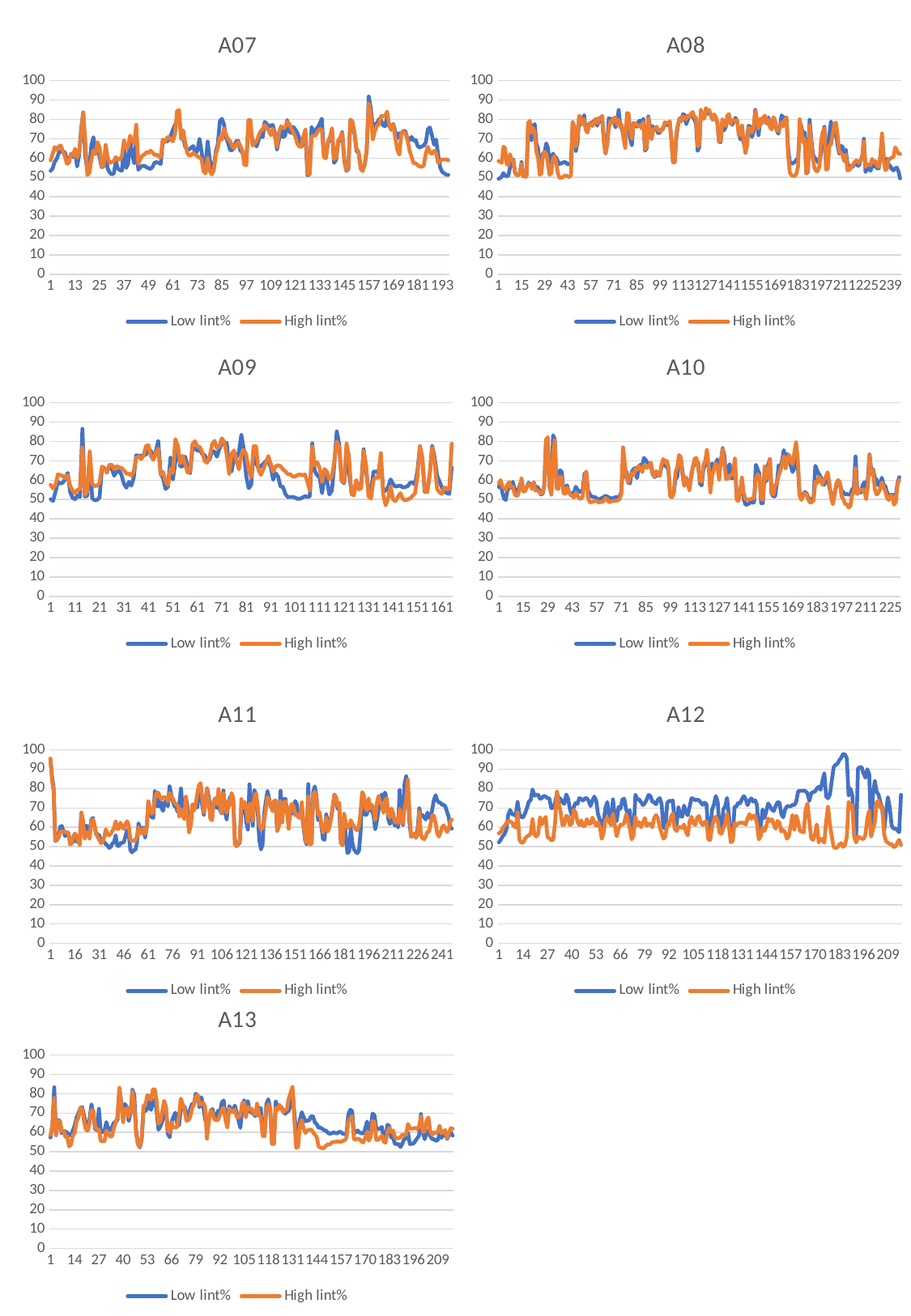

### Chart: A07
| Category | Low lint% | High lint% |
|---|---|---|
### Chart: A08
| Category | Low lint% | High lint% |
|---|---|---|
### Chart: A09
| Category | Low lint% | High lint% |
|---|---|---|
### Chart: A10
| Category | Low lint% | High lint% |
|---|---|---|
### Chart: A11
| Category | Low lint% | High lint% |
|---|---|---|
### Chart: A12
| Category | Low lint% | High lint% |
|---|---|---|
### Chart: A13
| Category | Low lint% | High lint% |
|---|---|---|

## Slide 4
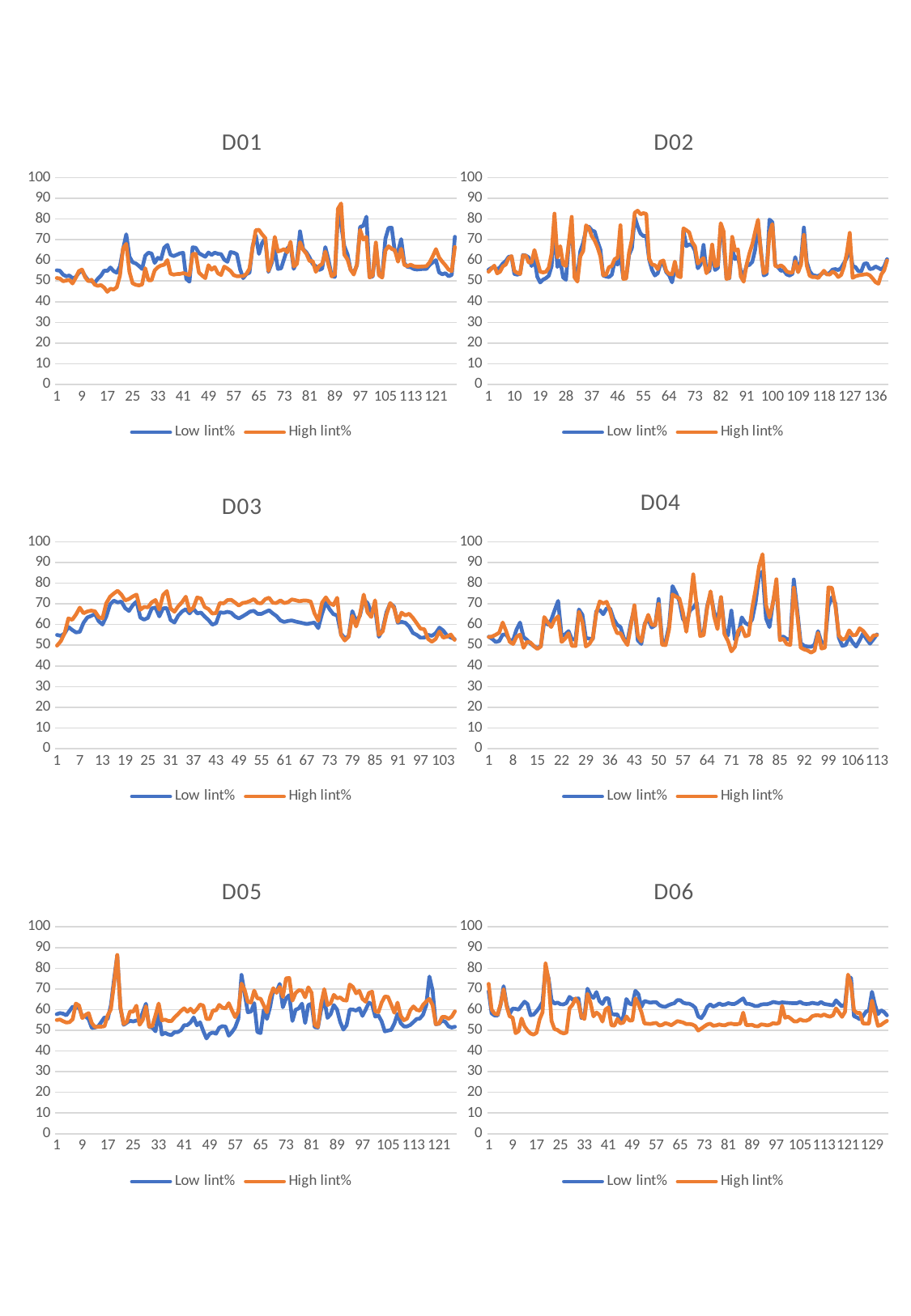

### Chart: D01
| Category | Low lint% | High lint% |
|---|---|---|
### Chart: D02
| Category | Low lint% | High lint% |
|---|---|---|
### Chart: D03
| Category | Low lint% | High lint% |
|---|---|---|
### Chart: D04
| Category | Low lint% | High lint% |
|---|---|---|
### Chart: D05
| Category | Low lint% | High lint% |
|---|---|---|
### Chart: D06
| Category | Low lint% | High lint% |
|---|---|---|

## Slide 5
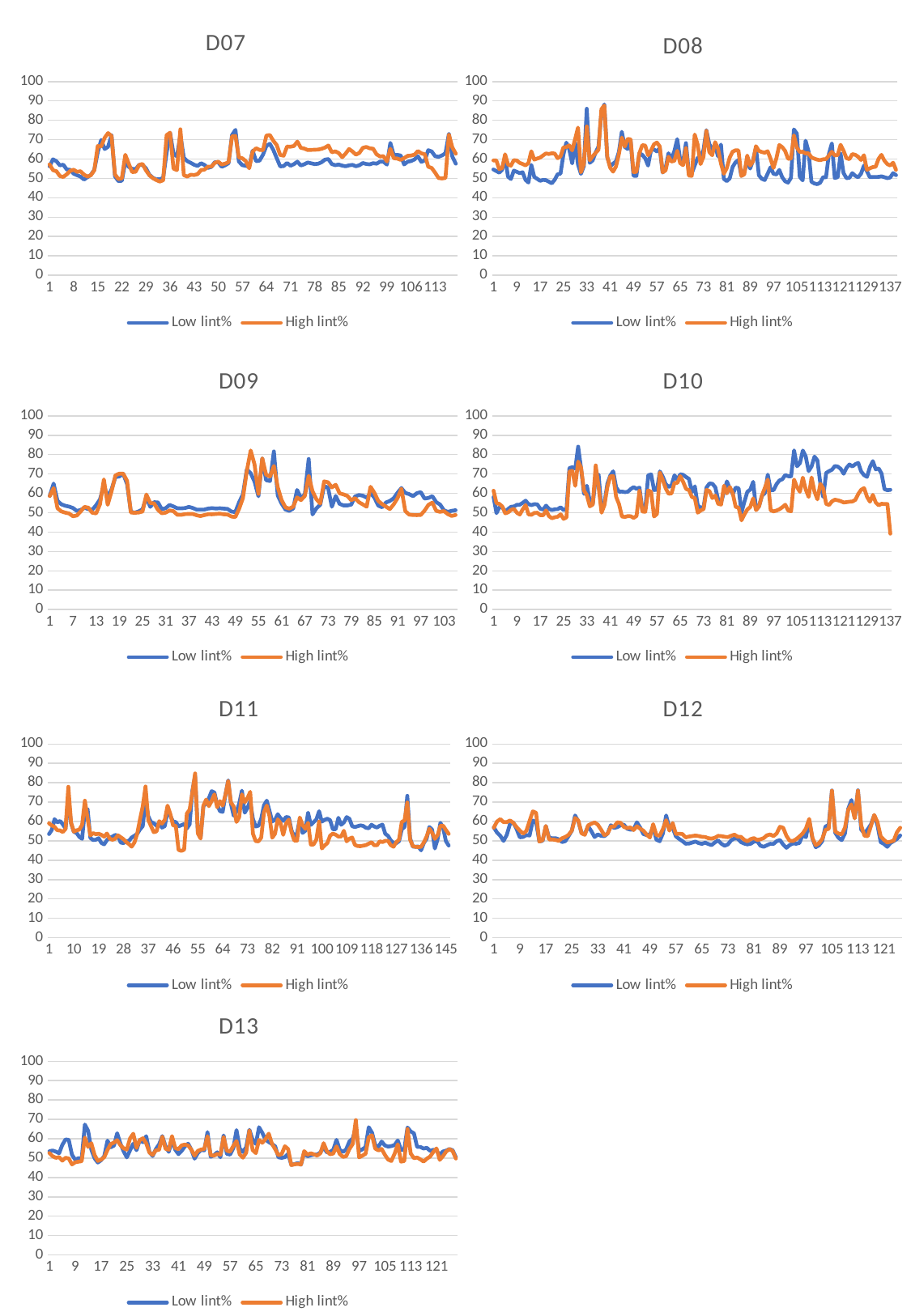

### Chart: D07
| Category | Low lint% | High lint% |
|---|---|---|
### Chart: D08
| Category | Low lint% | High lint% |
|---|---|---|
### Chart: D09
| Category | Low lint% | High lint% |
|---|---|---|
### Chart: D10
| Category | Low lint% | High lint% |
|---|---|---|
### Chart: D11
| Category | Low lint% | High lint% |
|---|---|---|
### Chart: D12
| Category | Low lint% | High lint% |
|---|---|---|
### Chart: D13
| Category | Low lint% | High lint% |
|---|---|---|

## Slide 6
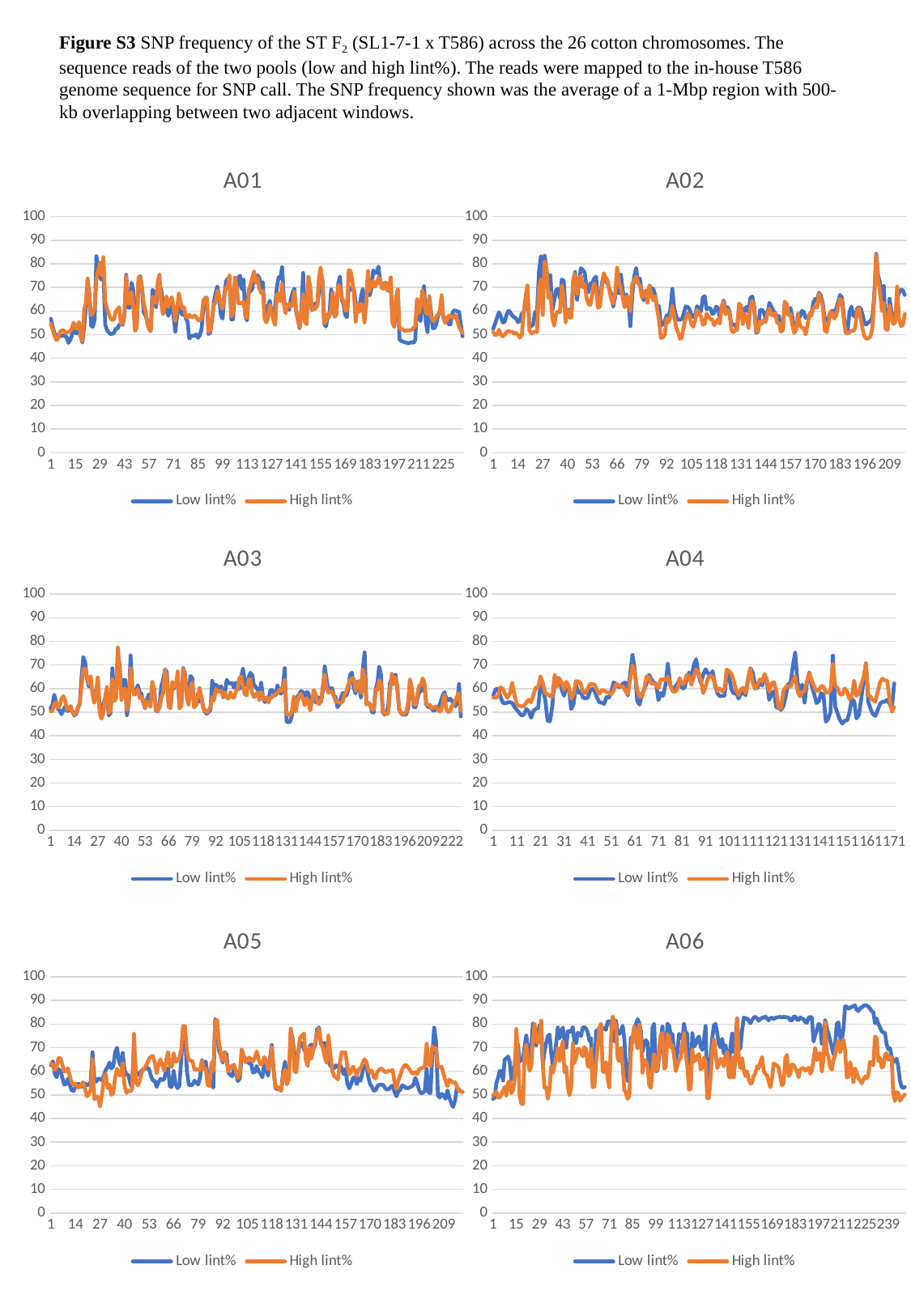

Figure S3 SNP frequency of the ST F2 (SL1-7-1 x T586) across the 26 cotton chromosomes. The sequence reads of the two pools (low and high lint%). The reads were mapped to the in-house T586 genome sequence for SNP call. The SNP frequency shown was the average of a 1-Mbp region with 500-kb overlapping between two adjacent windows.
### Chart: A01
| Category | Low lint% | High lint% |
|---|---|---|
### Chart: A02
| Category | Low lint% | High lint% |
|---|---|---|
### Chart: A03
| Category | Low lint% | High lint% |
|---|---|---|
### Chart: A04
| Category | Low lint% | High lint% |
|---|---|---|
### Chart: A05
| Category | Low lint% | High lint% |
|---|---|---|
### Chart: A06
| Category | Low lint% | High lint% |
|---|---|---|

## Slide 7
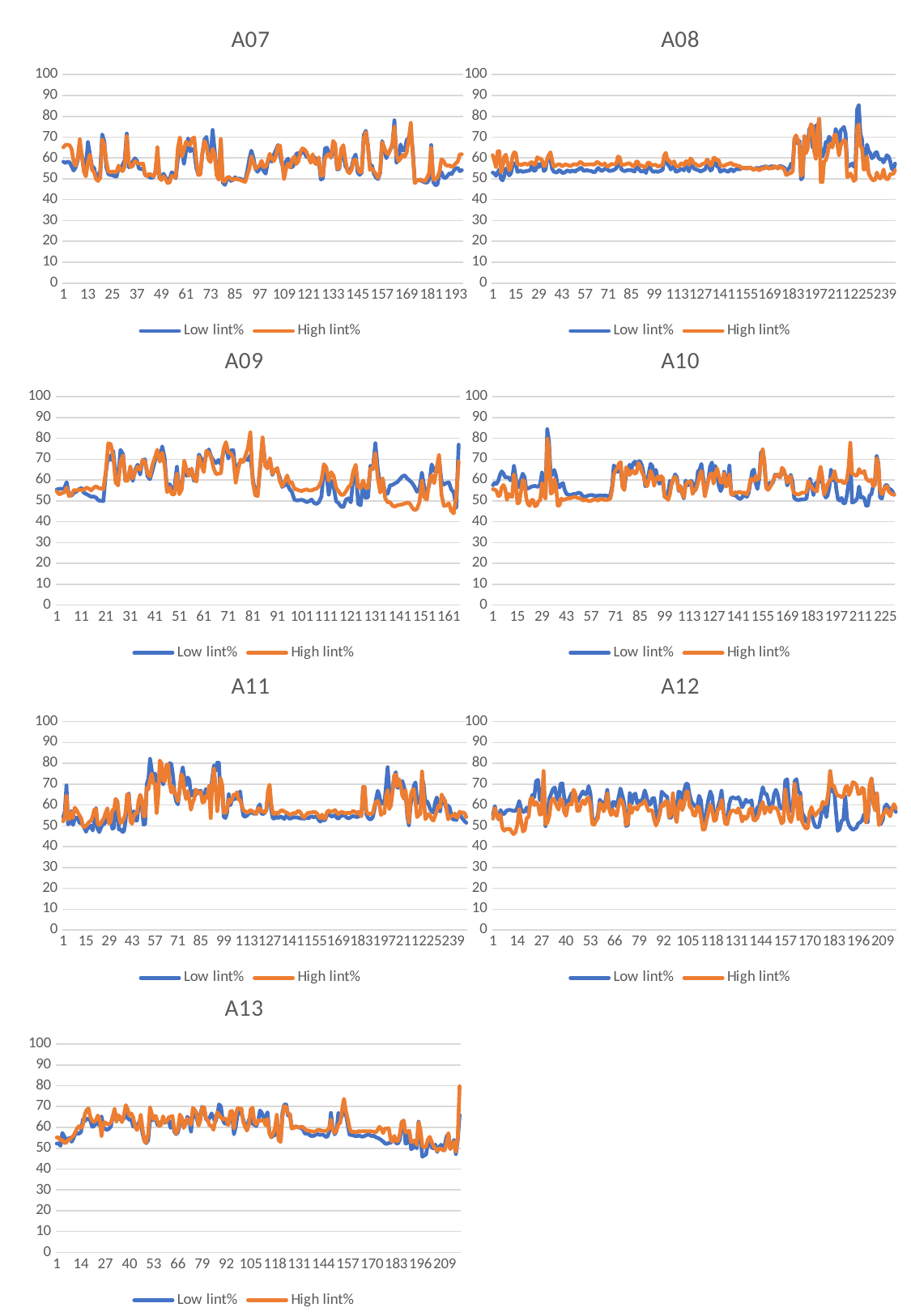

### Chart: A07
| Category | Low lint% | High lint% |
|---|---|---|
### Chart: A08
| Category | Low lint% | High lint% |
|---|---|---|
### Chart: A09
| Category | Low lint% | High lint% |
|---|---|---|
### Chart: A10
| Category | Low lint% | High lint% |
|---|---|---|
### Chart: A11
| Category | Low lint% | High lint% |
|---|---|---|
### Chart: A12
| Category | Low lint% | High lint% |
|---|---|---|
### Chart: A13
| Category | Low lint% | High lint% |
|---|---|---|

## Slide 8
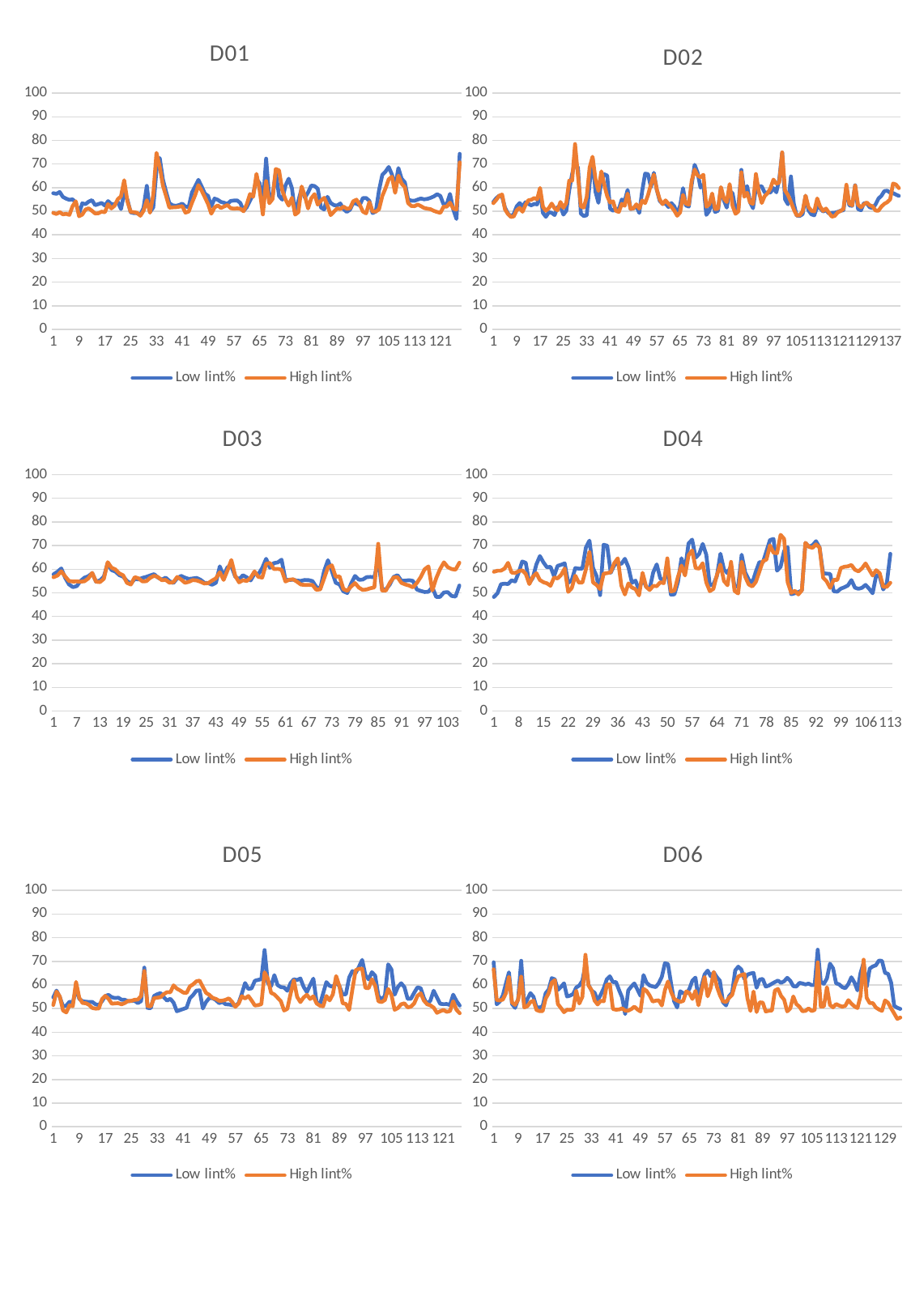

### Chart: D01
| Category | Low lint% | High lint% |
|---|---|---|
### Chart: D02
| Category | Low lint% | High lint% |
|---|---|---|
### Chart: D03
| Category | Low lint% | High lint% |
|---|---|---|
### Chart: D04
| Category | Low lint% | High lint% |
|---|---|---|
### Chart: D05
| Category | Low lint% | High lint% |
|---|---|---|
### Chart: D06
| Category | Low lint% | High lint% |
|---|---|---|

## Slide 9
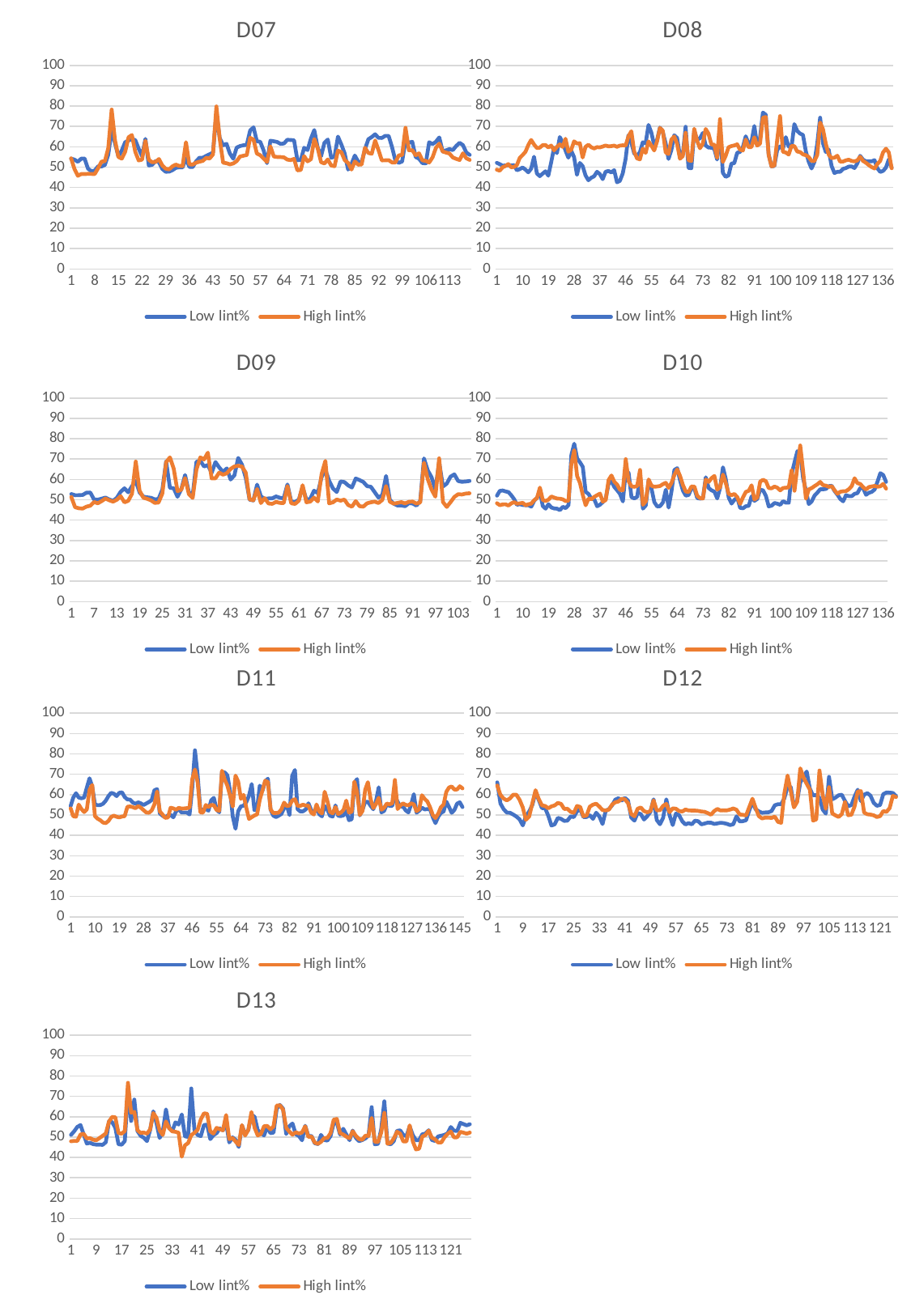

### Chart: D07
| Category | Low lint% | High lint% |
|---|---|---|
### Chart: D08
| Category | Low lint% | High lint% |
|---|---|---|
### Chart: D09
| Category | Low lint% | High lint% |
|---|---|---|
### Chart: D10
| Category | Low lint% | High lint% |
|---|---|---|
### Chart: D11
| Category | Low lint% | High lint% |
|---|---|---|
### Chart: D12
| Category | Low lint% | High lint% |
|---|---|---|
### Chart: D13
| Category | Low lint% | High lint% |
|---|---|---|

## Slide 10
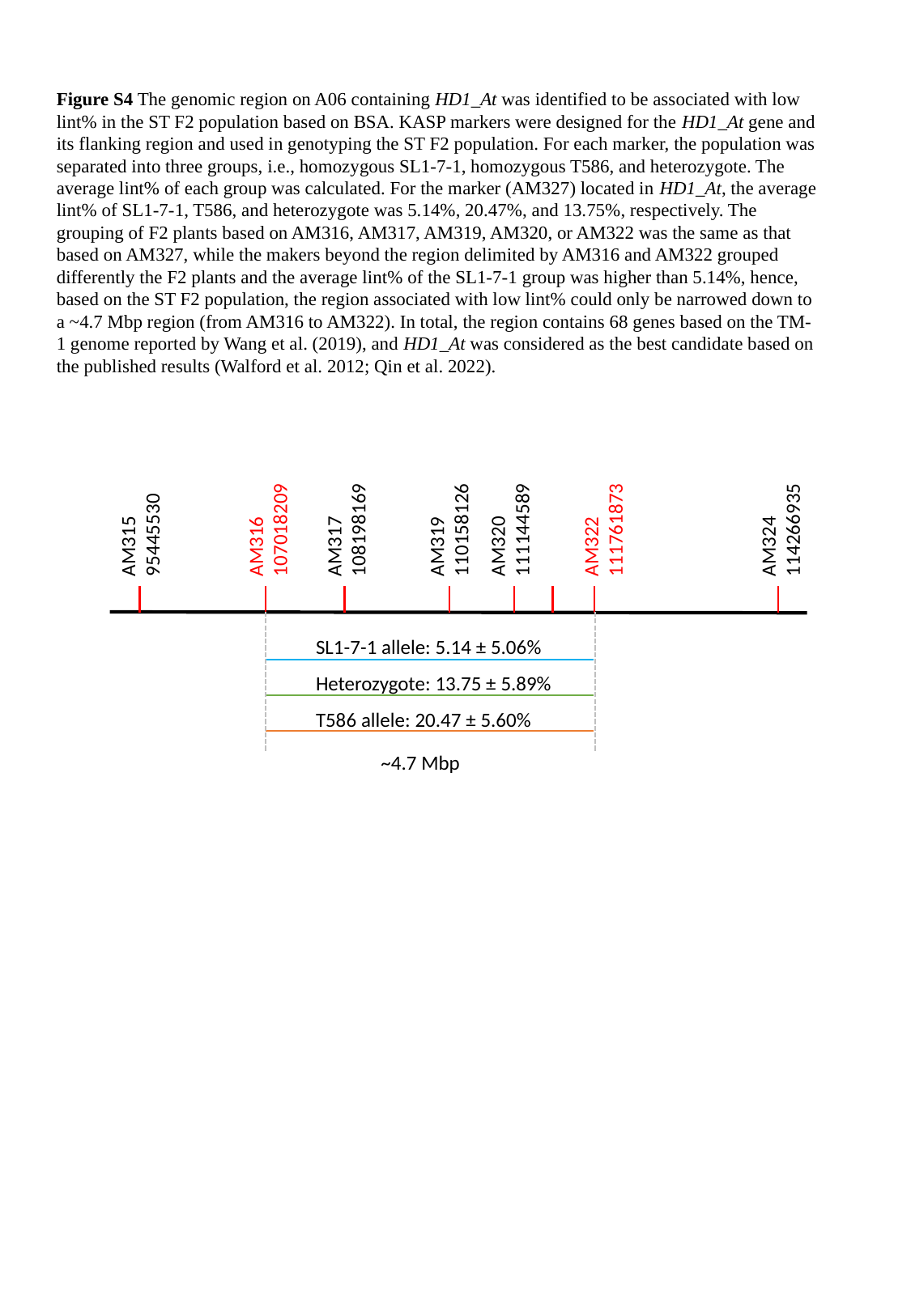

Figure S4 The genomic region on A06 containing HD1_At was identified to be associated with low lint% in the ST F2 population based on BSA. KASP markers were designed for the HD1_At gene and its flanking region and used in genotyping the ST F2 population. For each marker, the population was separated into three groups, i.e., homozygous SL1-7-1, homozygous T586, and heterozygote. The average lint% of each group was calculated. For the marker (AM327) located in HD1_At, the average lint% of SL1-7-1, T586, and heterozygote was 5.14%, 20.47%, and 13.75%, respectively. The grouping of F2 plants based on AM316, AM317, AM319, AM320, or AM322 was the same as that based on AM327, while the makers beyond the region delimited by AM316 and AM322 grouped differently the F2 plants and the average lint% of the SL1-7-1 group was higher than 5.14%, hence, based on the ST F2 population, the region associated with low lint% could only be narrowed down to a ~4.7 Mbp region (from AM316 to AM322). In total, the region contains 68 genes based on the TM-1 genome reported by Wang et al. (2019), and HD1_At was considered as the best candidate based on the published results (Walford et al. 2012; Qin et al. 2022).
AM317
108198169
AM319
110158126
AM320
111144589
AM324
114266935
AM322
111761873
AM316
107018209
AM315
95445530
SL1-7-1 allele: 5.14 ± 5.06%
Heterozygote: 13.75 ± 5.89%
T586 allele: 20.47 ± 5.60%
~4.7 Mbp

## Slide 11
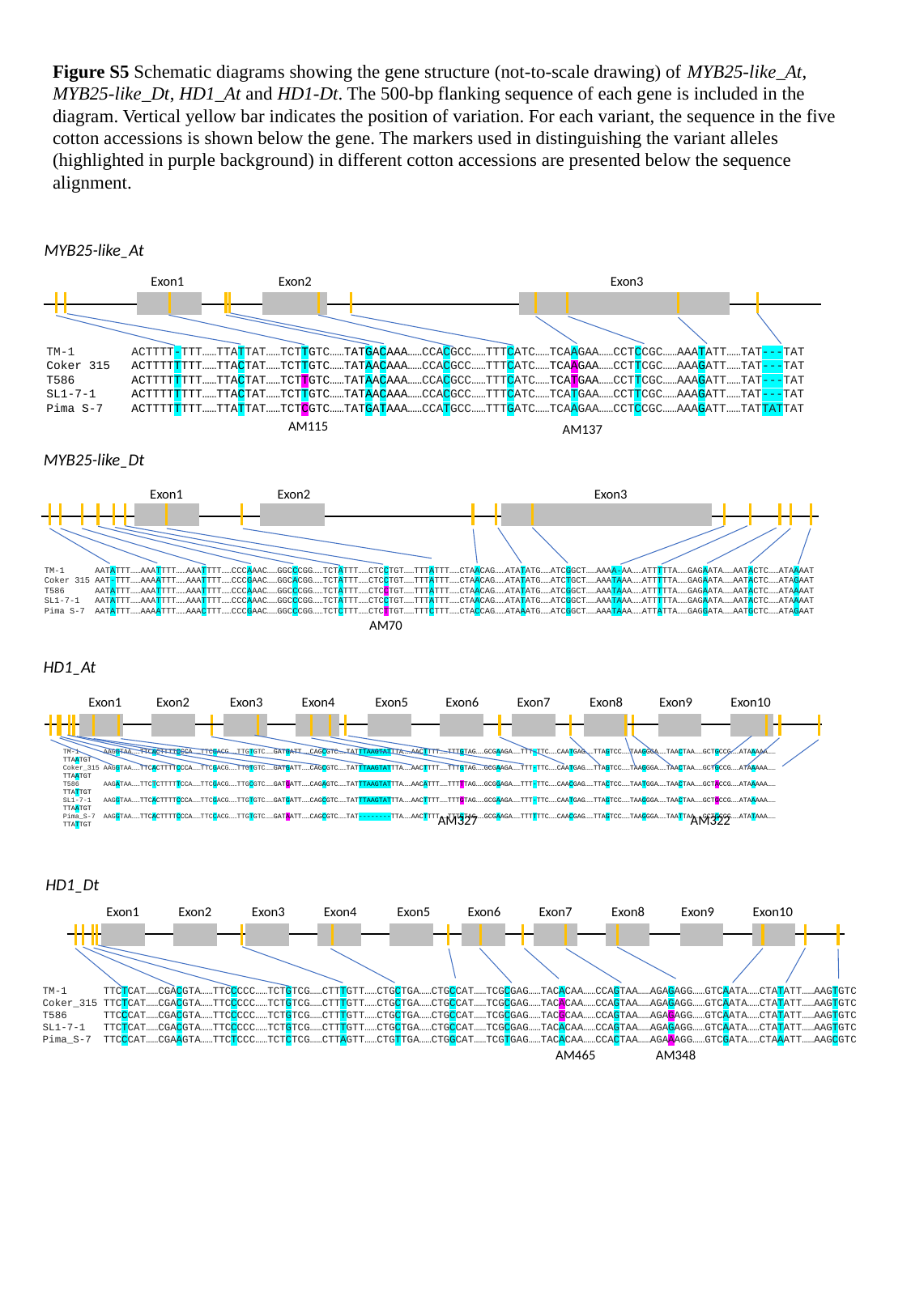

Figure S5 Schematic diagrams showing the gene structure (not-to-scale drawing) of MYB25-like_At, MYB25-like_Dt, HD1_At and HD1-Dt. The 500-bp flanking sequence of each gene is included in the diagram. Vertical yellow bar indicates the position of variation. For each variant, the sequence in the five cotton accessions is shown below the gene. The markers used in distinguishing the variant alleles (highlighted in purple background) in different cotton accessions are presented below the sequence alignment.
MYB25-like_At
Exon1
Exon2
Exon3
TM-1 ACTTTT-TTT……TTATTAT……TCTTGTC……TATGACAAA……CCACGCC……TTTCATC……TCAAGAA……CCTCCGC……AAATATT……TAT---TAT
Coker 315 ACTTTTTTTT……TTACTAT……TCTTGTC……TATAACAAA……CCACGCC……TTTCATC……TCAAGAA……CCTTCGC……AAAGATT……TAT---TAT
T586 ACTTTTTTTT……TTACTAT……TCTTGTC……TATAACAAA……CCACGCC……TTTCATC……TCATGAA……CCTTCGC……AAAGATT……TAT---TAT
SL1-7-1 ACTTTTTTTT……TTACTAT……TCTTGTC……TATAACAAA……CCACGCC……TTTCATC……TCATGAA……CCTTCGC……AAAGATT……TAT---TAT
Pima S-7 ACTTTTTTTT……TTATTAT……TCTCGTC……TATGATAAA……CCATGCC……TTTGATC……TCAAGAA……CCTCCGC……AAAGATT……TATTATTAT
AM115
AM137
MYB25-like_Dt
Exon1
Exon2
Exon3
TM-1 AATATTT……AAATTTT……AAATTTT……CCCAAAC……GGCCCGG……TCTATTT……CTCCTGT……TTTATTT……CTAACAG……ATATATG……ATCGGCT……AAAA-AA……ATTTTTA……GAGAATA……AATACTC……ATAAAAT
Coker 315 AAT-TTT……AAAATTT……AAATTTT……CCCGAAC……GGCACGG……TCTATTT……CTCCTGT……TTTATTT……CTAACAG……ATATATG……ATCTGCT……AAATAAA……ATTTTTA……GAGAATA……AATACTC……ATAGAAT
T586 AATATTT……AAATTTT……AAATTTT……CCCAAAC……GGCCCGG……TCTATTT……CTCCTGT……TTTATTT……CTAACAG……ATATATG……ATCGGCT……AAATAAA……ATTTTTA……GAGAATA……AATACTC……ATAAAAT
SL1-7-1 AATATTT……AAATTTT……AAATTTT……CCCAAAC……GGCCCGG……TCTATTT……CTCCTGT……TTTATTT……CTAACAG……ATATATG……ATCGGCT……AAATAAA……ATTTTTA……GAGAATA……AATACTC……ATAAAAT
Pima S-7 AATATTT……AAAATTT……AAACTTT……CCCGAAC……GGCCCGG……TCTCTTT……CTCTTGT……TTTCTTT……CTACCAG……ATAAATG……ATCGGCT……AAATAAA……ATTATTA……GAGGATA……AATGCTC……ATAGAAT
AM70
HD1_At
Exon1
Exon2
Exon3
Exon4
Exon5
Exon6
Exon7
Exon8
Exon9
Exon10
TM-1 AAGGTAA……TTCACTTTTCCCA……TTCGACG……TTGTGTC……GATGATT……CAGCGTC……TATTTAAGTATTTA……AACTTTT……TTTGTAG……GCGAAGA……TTT-TTC……CAATGAG……TTAGTCC……TAAGGGA……TAACTAA……GCTGCCG……ATAAAAA……TTAATGT
Coker_315 AAGGTAA……TTCACTTTTCCCA……TTCGACG……TTGTGTC……GATGATT……CAGCGTC……TATTTAAGTATTTA……AACTTTT……TTTGTAG……GCGAAGA……TTT-TTC……CAATGAG……TTAGTCC……TAAGGGA……TAACTAA……GCTGCCG……ATAAAAA……TTAATGT
T586 AAGATAA……TTCTCTTTTTCCA……TTCGACG……TTGCGTC……GATGATT……CAGAGTC……TATTTAAGTATTTA……AACATTT……TTTTTAG……GCGGAGA……TTT-TTC……CAACGAG……TTACTCC……TAATGGA……TAACTAA……GCTACCG……ATAAAAA……TTATTGT
SL1-7-1 AAGGTAA……TTCACTTTTCCCA……TTCGACG……TTGTGTC……GATGATT……CAGCGTC……TATTTAAGTATTTA……AACTTTT……TTTGTAG……GCGAAGA……TTT-TTC……CAATGAG……TTAGTCC……TAAGGGA……TAACTAA……GCTGCCG……ATAAAAA……TTAATGT
Pima_S-7 AAGGTAA……TTCACTTTTCCCA……TTCCACG……TTGTGTC……GATAATT……CAGCGTC……TAT--------TTA……AACTTTT……TTTGTAG……GCGAAGA……TTTTTTC……CAACGAG……TTAGTCC……TAAGGGA……TAATTAA……GCTGCCG……ATATAAA……TTATTGT
AM294
AM327
AM322
HD1_Dt
Exon1
Exon2
Exon3
Exon4
Exon5
Exon6
Exon7
Exon8
Exon9
Exon10
TM-1 TTCTCAT……CGACGTA……TTCCCCC……TCTGTCG……CTTTGTT……CTGCTGA……CTGCCAT……TCGCGAG……TACACAA……CCAGTAA……AGAGAGG……GTCAATA……CTATATT……AAGTGTC
Coker_315 TTCTCAT……CGACGTA……TTCCCCC……TCTGTCG……CTTTGTT……CTGCTGA……CTGCCAT……TCGCGAG……TACACAA……CCAGTAA……AGAGAGG……GTCAATA……CTATATT……AAGTGTC
T586 TTCCCAT……CGACGTA……TTCCCCC……TCTGTCG……CTTTGTT……CTGCTGA……CTGCCAT……TCGCGAG……TACGCAA……CCAGTAA……AGAGAGG……GTCAATA……CTATATT……AAGTGTC
SL1-7-1 TTCTCAT……CGACGTA……TTCCCCC……TCTGTCG……CTTTGTT……CTGCTGA……CTGCCAT……TCGCGAG……TACACAA……CCAGTAA……AGAGAGG……GTCAATA……CTATATT……AAGTGTC
Pima_S-7 TTCCCAT……CGAAGTA……TTCTCCC……TCTCTCG……CTTAGTT……CTGTTGA……CTGGCAT……TCGTGAG……TACACAA……CCACTAA……AGAAAGG……GTCGATA……CTAAATT……AAGCGTC
AM465
AM348

## Slide 12
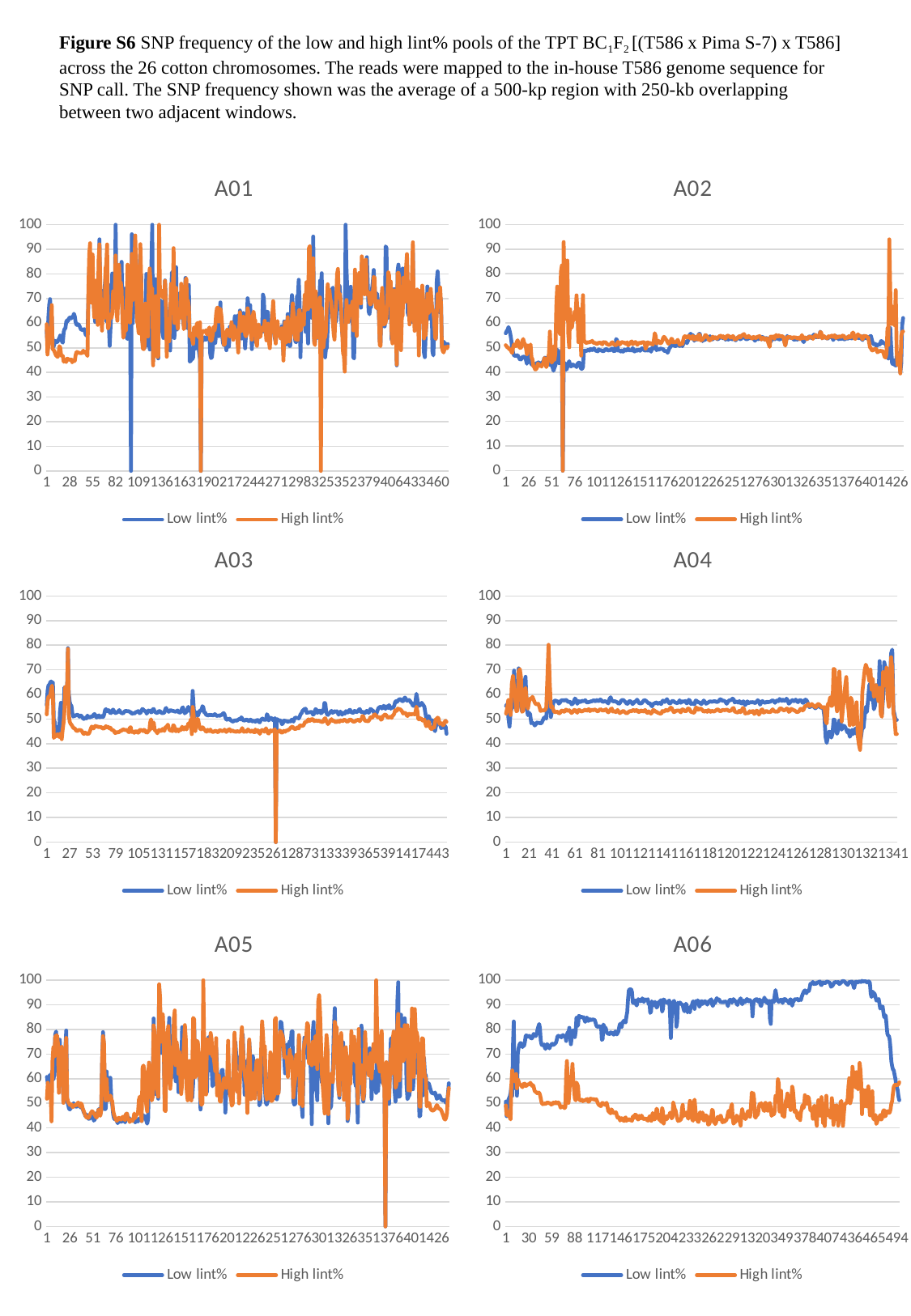

Figure S6 SNP frequency of the low and high lint% pools of the TPT BC1F2 [(T586 x Pima S-7) x T586] across the 26 cotton chromosomes. The reads were mapped to the in-house T586 genome sequence for SNP call. The SNP frequency shown was the average of a 500-kp region with 250-kb overlapping between two adjacent windows.
### Chart: A02
| Category | Low lint% | High lint% |
|---|---|---|
### Chart: A01
| Category | Low lint% | High lint% |
|---|---|---|
### Chart: A03
| Category | Low lint% | High lint% |
|---|---|---|
### Chart: A04
| Category | Low lint% | High lint% |
|---|---|---|
### Chart: A05
| Category | Low lint% | High lint% |
|---|---|---|
### Chart: A06
| Category | Low lint% | High lint% |
|---|---|---|

## Slide 13
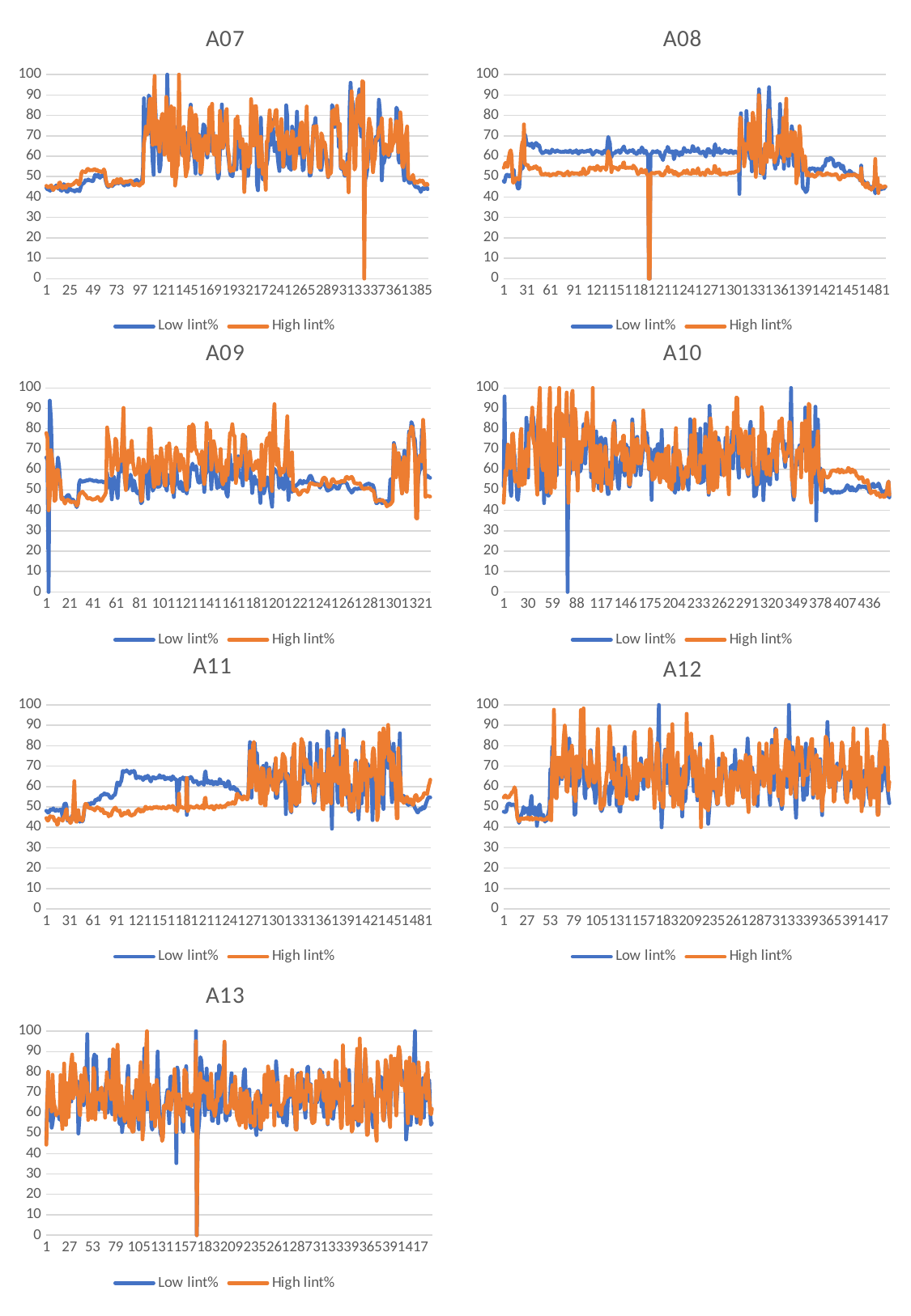

### Chart: A07
| Category | Low lint% | High lint% |
|---|---|---|
### Chart: A08
| Category | Low lint% | High lint% |
|---|---|---|
### Chart: A09
| Category | Low lint% | High lint% |
|---|---|---|
### Chart: A10
| Category | Low lint% | High lint% |
|---|---|---|
### Chart: A11
| Category | Low lint% | High lint% |
|---|---|---|
### Chart: A12
| Category | Low lint% | High lint% |
|---|---|---|
### Chart: A13
| Category | Low lint% | High lint% |
|---|---|---|

## Slide 14
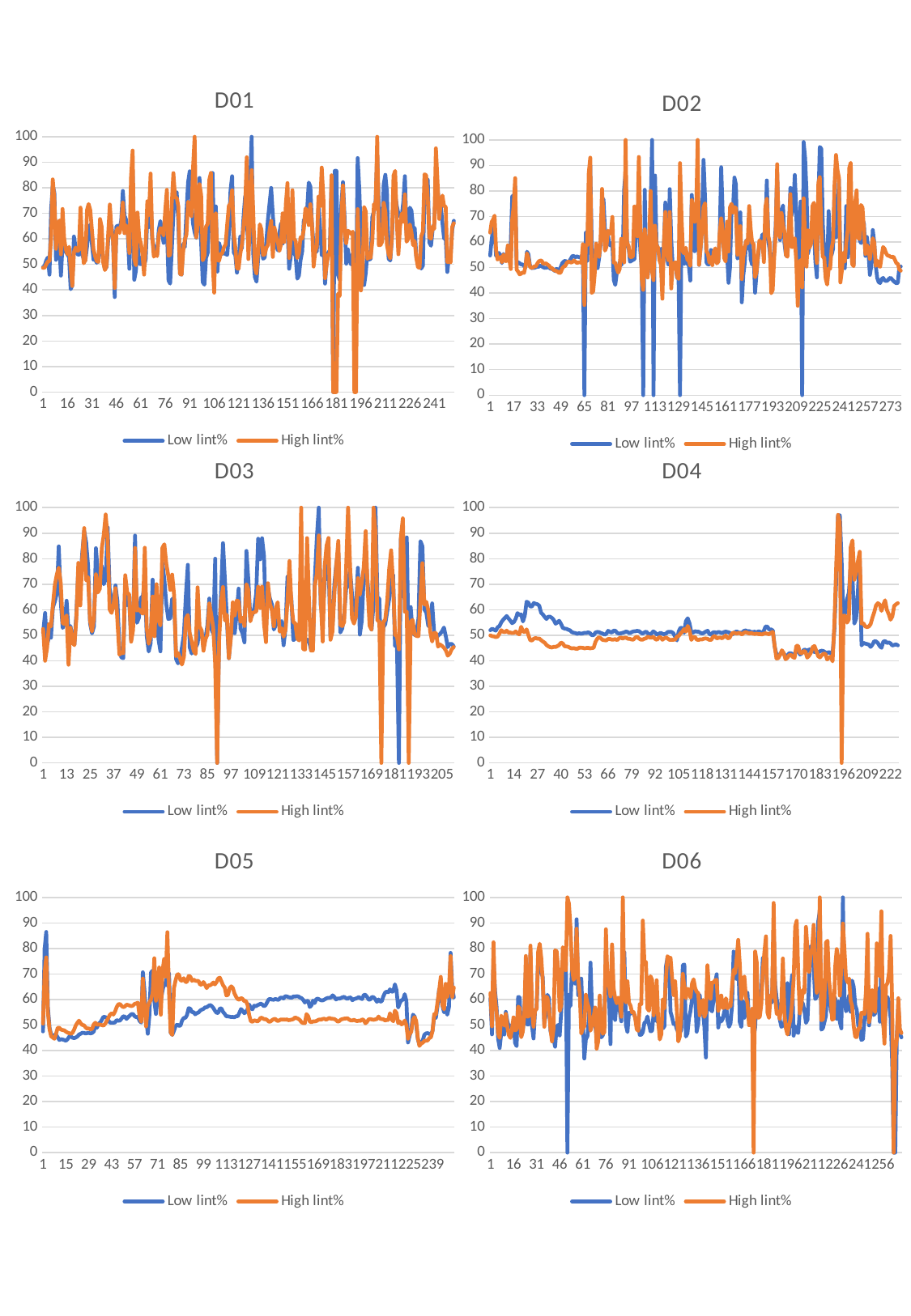

### Chart: D01
| Category | Low lint% | High lint% |
|---|---|---|
### Chart: D02
| Category | Low lint% | High lint% |
|---|---|---|
### Chart: D03
| Category | Low lint% | High lint% |
|---|---|---|
### Chart: D04
| Category | Low lint% | High lint% |
|---|---|---|
### Chart: D05
| Category | Low lint% | High lint% |
|---|---|---|
### Chart: D06
| Category | Low lint% | High lint% |
|---|---|---|

## Slide 15
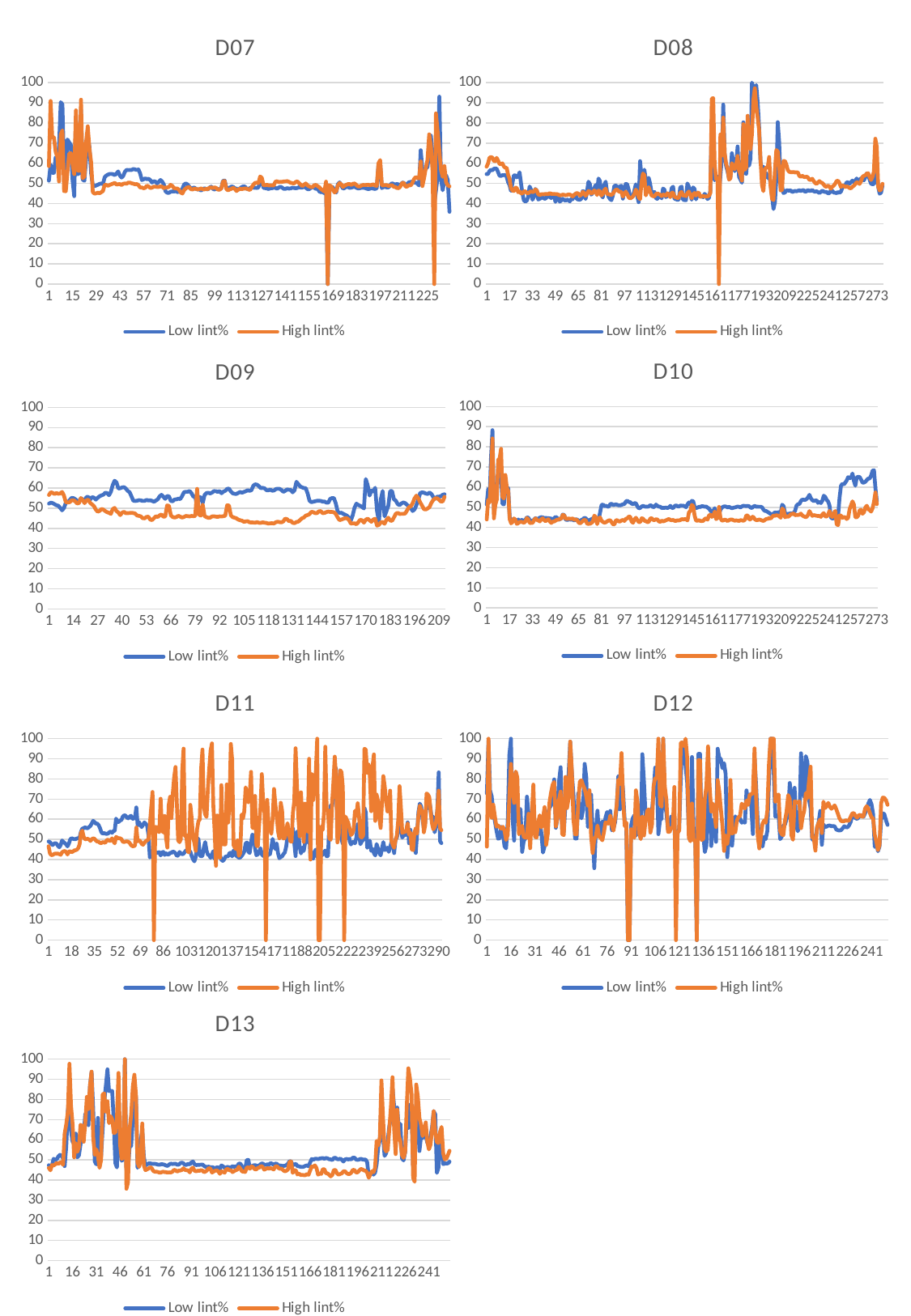

### Chart: D07
| Category | Low lint% | High lint% |
|---|---|---|
### Chart: D08
| Category | Low lint% | High lint% |
|---|---|---|
### Chart: D10
| Category | Low lint% | High lint% |
|---|---|---|
### Chart: D09
| Category | Low lint% | High lint% |
|---|---|---|
### Chart: D11
| Category | Low lint% | High lint% |
|---|---|---|
### Chart: D12
| Category | Low lint% | High lint% |
|---|---|---|
### Chart: D13
| Category | Low lint% | High lint% |
|---|---|---|

## Slide 16
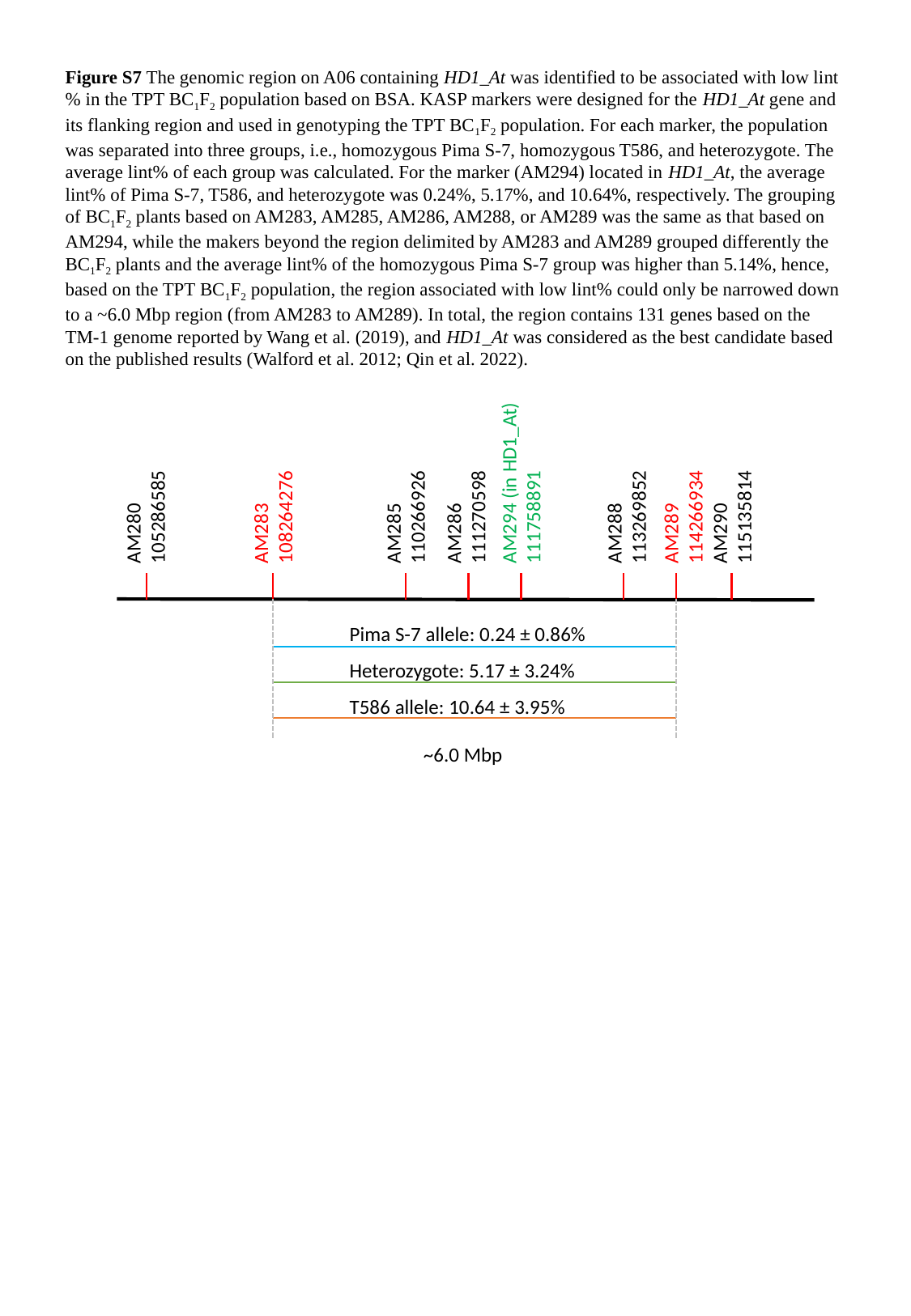

Figure S7 The genomic region on A06 containing HD1_At was identified to be associated with low lint% in the TPT BC1F2 population based on BSA. KASP markers were designed for the HD1_At gene and its flanking region and used in genotyping the TPT BC1F2 population. For each marker, the population was separated into three groups, i.e., homozygous Pima S-7, homozygous T586, and heterozygote. The average lint% of each group was calculated. For the marker (AM294) located in HD1_At, the average lint% of Pima S-7, T586, and heterozygote was 0.24%, 5.17%, and 10.64%, respectively. The grouping of BC1F2 plants based on AM283, AM285, AM286, AM288, or AM289 was the same as that based on AM294, while the makers beyond the region delimited by AM283 and AM289 grouped differently the BC1F2 plants and the average lint% of the homozygous Pima S-7 group was higher than 5.14%, hence, based on the TPT BC1F2 population, the region associated with low lint% could only be narrowed down to a ~6.0 Mbp region (from AM283 to AM289). In total, the region contains 131 genes based on the TM-1 genome reported by Wang et al. (2019), and HD1_At was considered as the best candidate based on the published results (Walford et al. 2012; Qin et al. 2022).
AM294 (in HD1_At)
111758891
AM285
110266926
AM286
111270598
AM288
113269852
AM290
115135814
AM280
105286585
AM289
114266934
AM283
108264276
Pima S-7 allele: 0.24 ± 0.86%
Heterozygote: 5.17 ± 3.24%
T586 allele: 10.64 ± 3.95%
~6.0 Mbp

## Slide 17
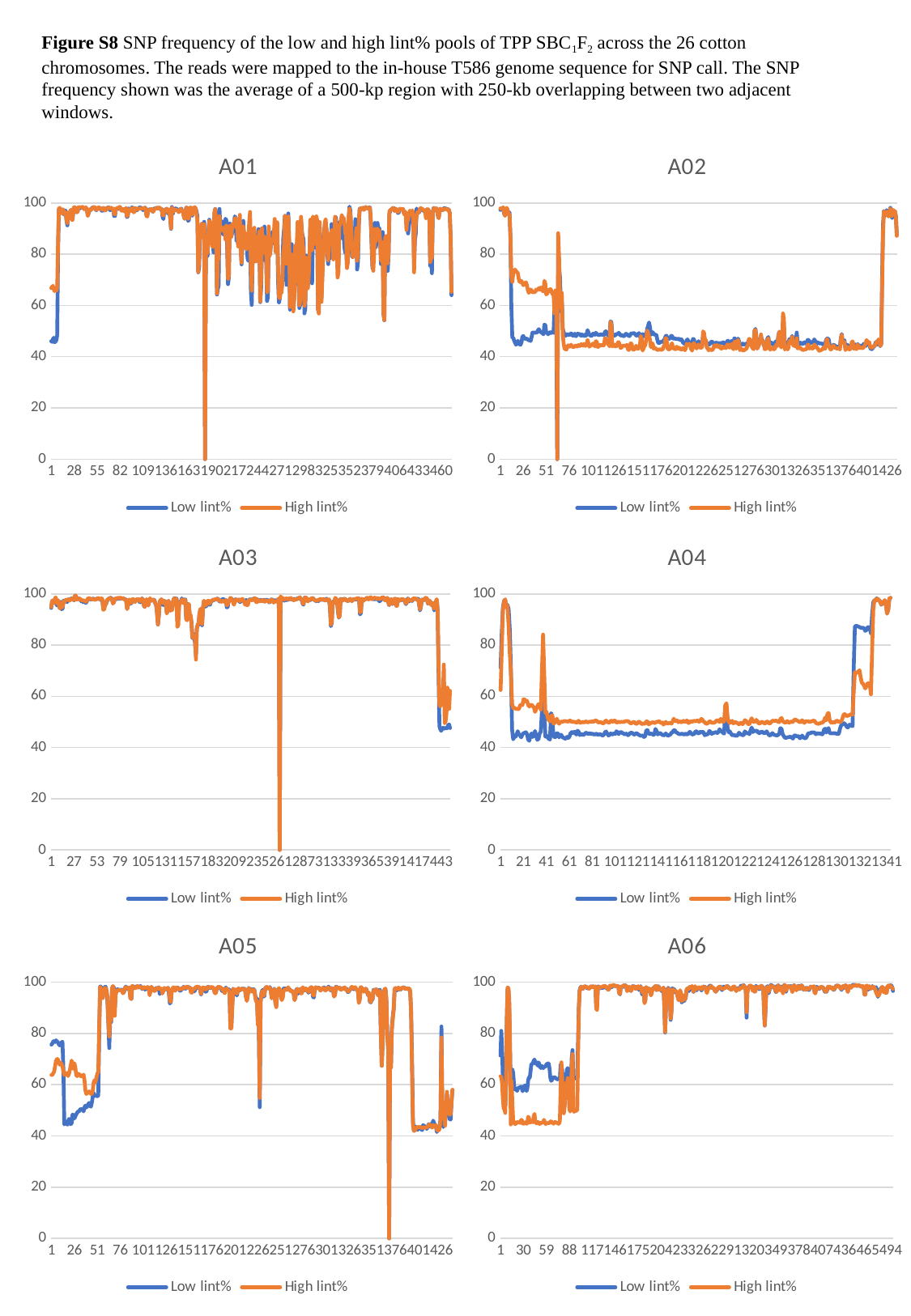

Figure S8 SNP frequency of the low and high lint% pools of TPP SBC1F2 across the 26 cotton chromosomes. The reads were mapped to the in-house T586 genome sequence for SNP call. The SNP frequency shown was the average of a 500-kp region with 250-kb overlapping between two adjacent windows.
### Chart: A01
| Category | Low lint% | High lint% |
|---|---|---|
### Chart: A02
| Category | Low lint% | High lint% |
|---|---|---|
### Chart: A03
| Category | Low lint% | High lint% |
|---|---|---|
### Chart: A04
| Category | Low lint% | High lint% |
|---|---|---|
### Chart: A05
| Category | Low lint% | High lint% |
|---|---|---|
### Chart: A06
| Category | Low lint% | High lint% |
|---|---|---|

## Slide 18
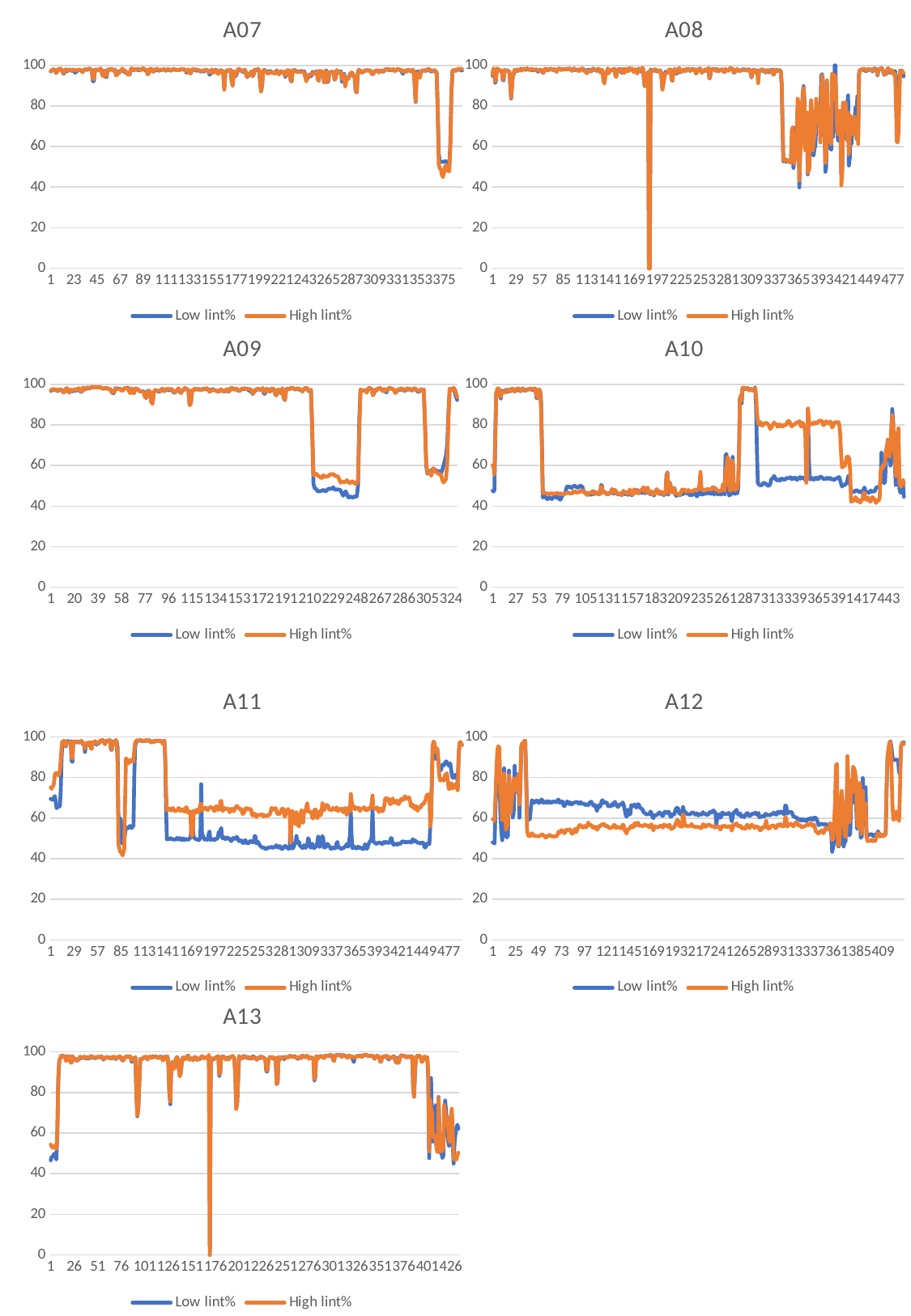

### Chart: A07
| Category | Low lint% | High lint% |
|---|---|---|
### Chart: A08
| Category | Low lint% | High lint% |
|---|---|---|
### Chart: A09
| Category | Low lint% | High lint% |
|---|---|---|
### Chart: A10
| Category | Low lint% | High lint% |
|---|---|---|
### Chart: A11
| Category | Low lint% | High lint% |
|---|---|---|
### Chart: A12
| Category | Low lint% | High lint% |
|---|---|---|
### Chart: A13
| Category | Low lint% | High lint% |
|---|---|---|

## Slide 19
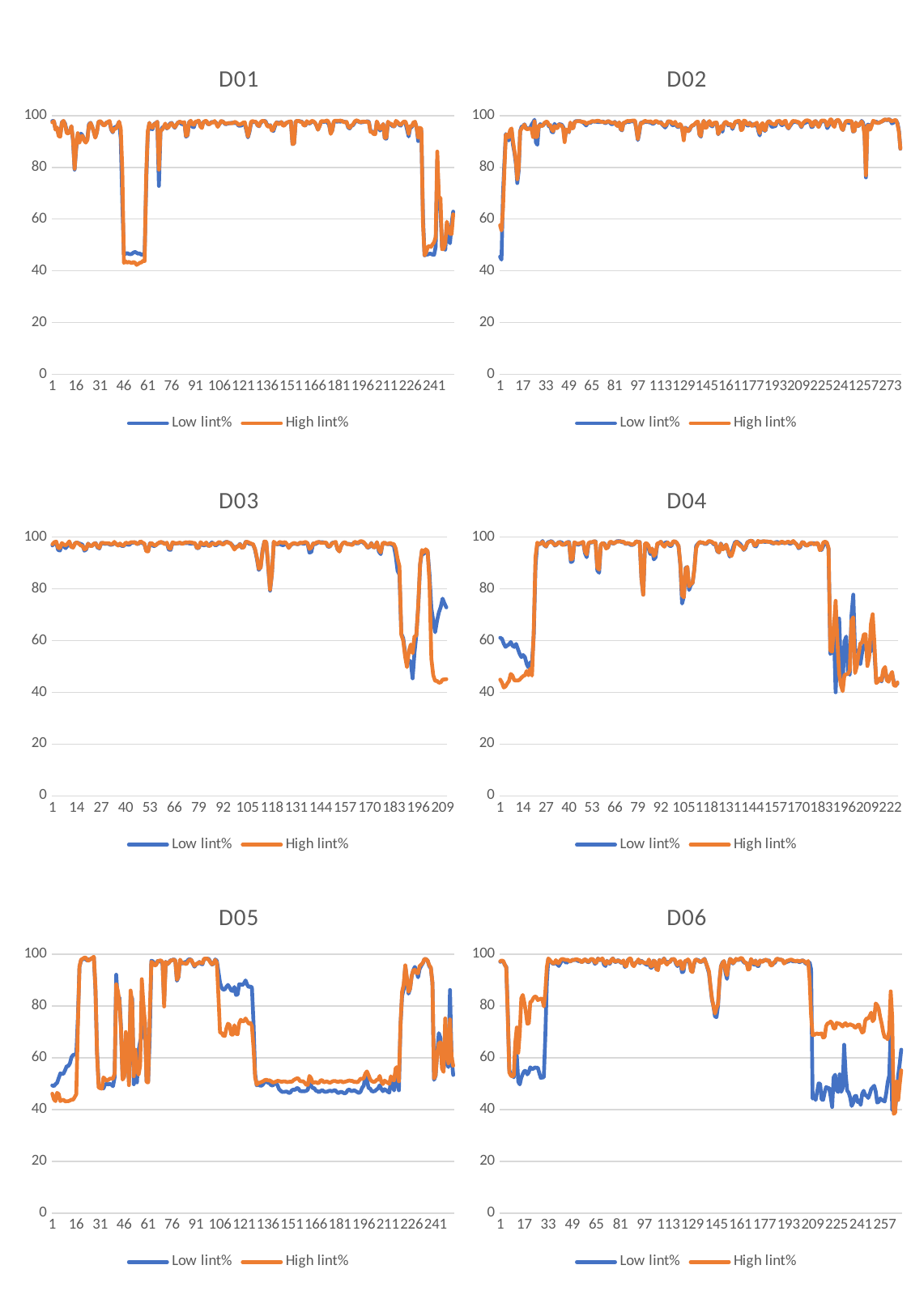

### Chart: D01
| Category | Low lint% | High lint% |
|---|---|---|
### Chart: D02
| Category | Low lint% | High lint% |
|---|---|---|
### Chart: D03
| Category | Low lint% | High lint% |
|---|---|---|
### Chart: D04
| Category | Low lint% | High lint% |
|---|---|---|
### Chart: D05
| Category | Low lint% | High lint% |
|---|---|---|
### Chart: D06
| Category | Low lint% | High lint% |
|---|---|---|

## Slide 20
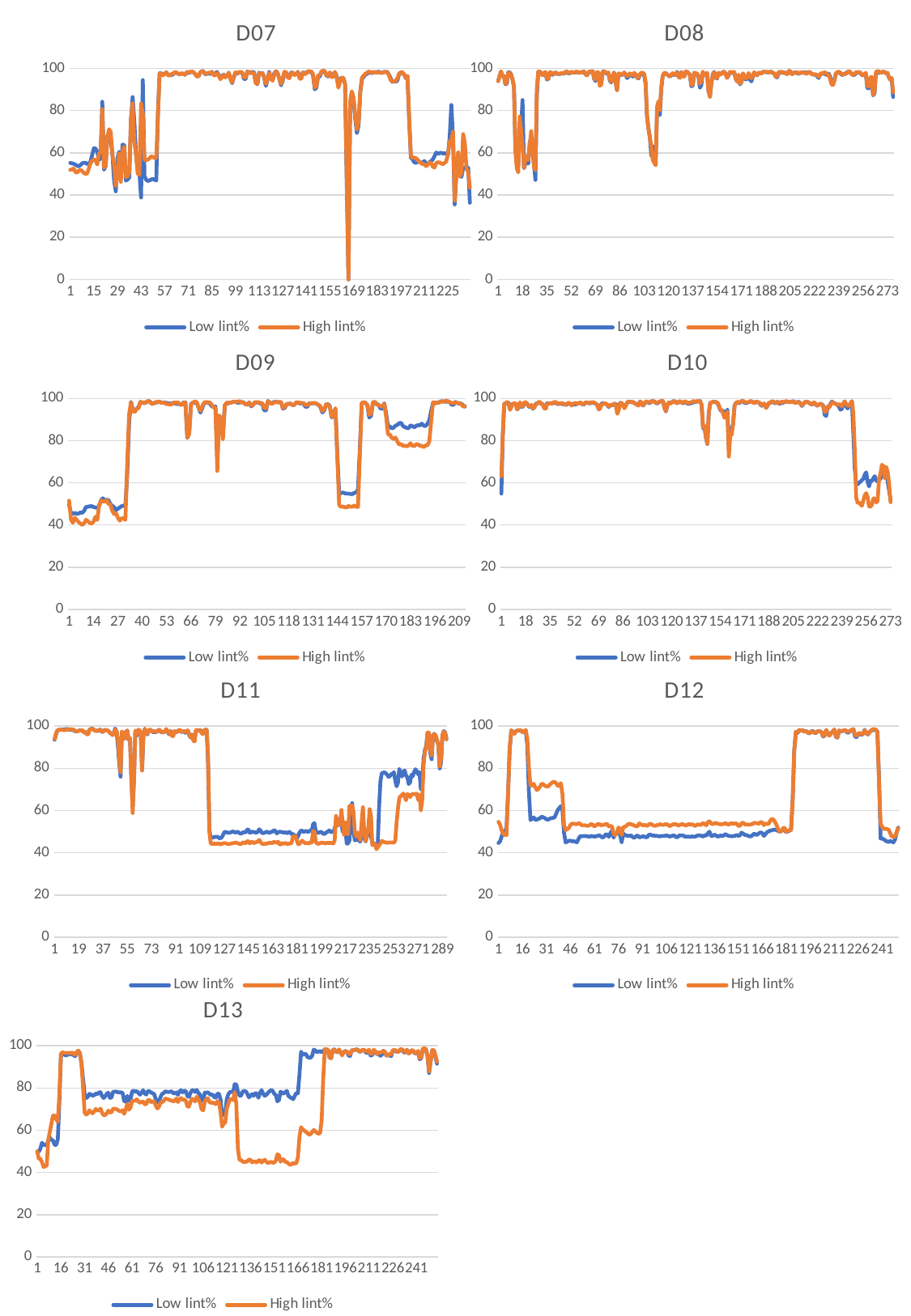

### Chart: D07
| Category | Low lint% | High lint% |
|---|---|---|
### Chart: D08
| Category | Low lint% | High lint% |
|---|---|---|
### Chart: D09
| Category | Low lint% | High lint% |
|---|---|---|
### Chart: D10
| Category | Low lint% | High lint% |
|---|---|---|
### Chart: D11
| Category | Low lint% | High lint% |
|---|---|---|
### Chart: D12
| Category | Low lint% | High lint% |
|---|---|---|
### Chart: D13
| Category | Low lint% | High lint% |
|---|---|---|

## Slide 21
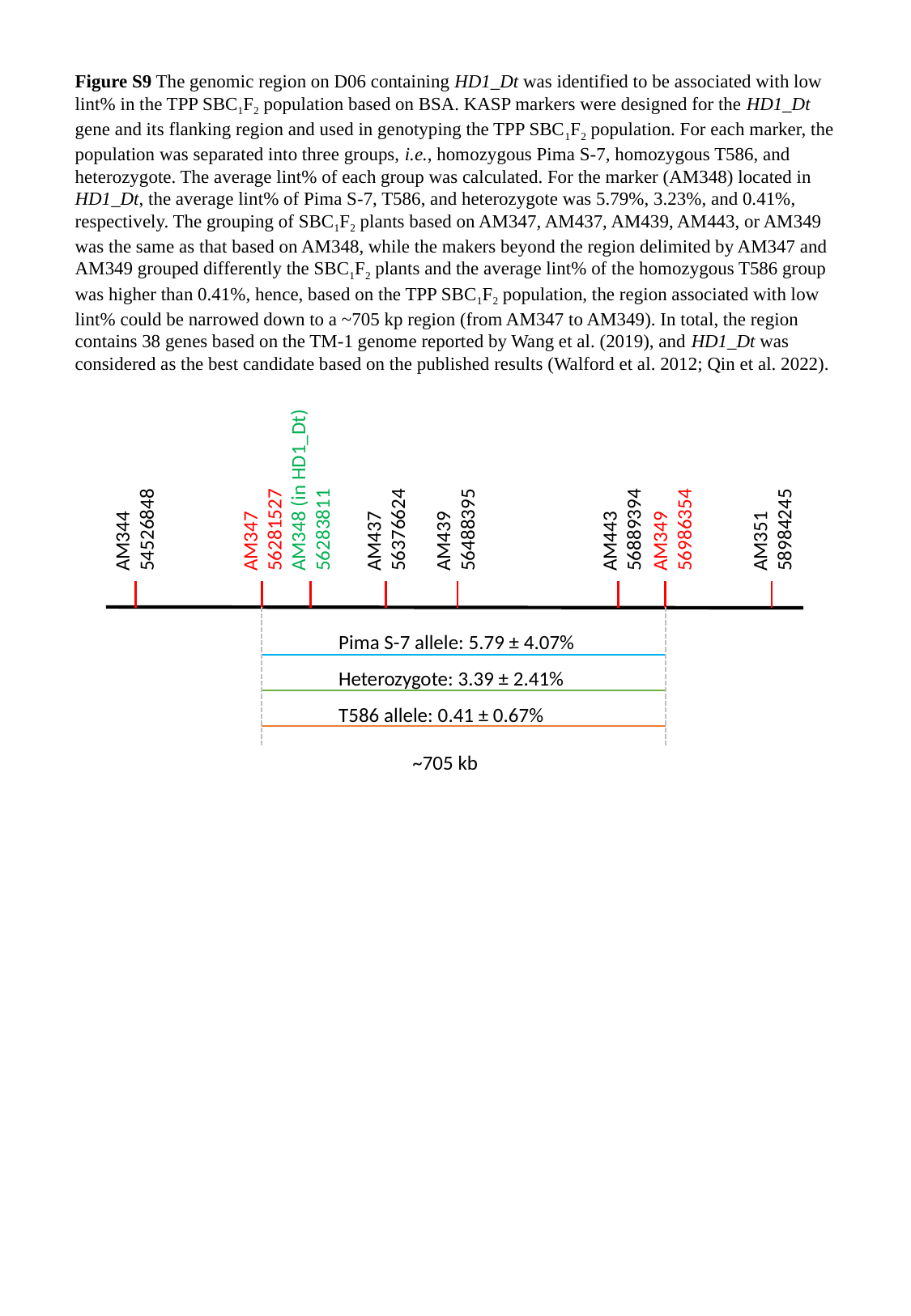

Figure S9 The genomic region on D06 containing HD1_Dt was identified to be associated with low lint% in the TPP SBC1F2 population based on BSA. KASP markers were designed for the HD1_Dt gene and its flanking region and used in genotyping the TPP SBC1F2 population. For each marker, the population was separated into three groups, i.e., homozygous Pima S-7, homozygous T586, and heterozygote. The average lint% of each group was calculated. For the marker (AM348) located in HD1_Dt, the average lint% of Pima S-7, T586, and heterozygote was 5.79%, 3.23%, and 0.41%, respectively. The grouping of SBC1F2 plants based on AM347, AM437, AM439, AM443, or AM349 was the same as that based on AM348, while the makers beyond the region delimited by AM347 and AM349 grouped differently the SBC1F2 plants and the average lint% of the homozygous T586 group was higher than 0.41%, hence, based on the TPP SBC1F2 population, the region associated with low lint% could be narrowed down to a ~705 kp region (from AM347 to AM349). In total, the region contains 38 genes based on the TM-1 genome reported by Wang et al. (2019), and HD1_Dt was considered as the best candidate based on the published results (Walford et al. 2012; Qin et al. 2022).
AM348 (in HD1_Dt)
56283811
AM437
56376624
AM439
56488395
AM443
56889394
AM351
58984245
AM344
54526848
AM349
56986354
AM347
56281527
Pima S-7 allele: 5.79 ± 4.07%
Heterozygote: 3.39 ± 2.41%
T586 allele: 0.41 ± 0.67%
~705 kb

## Slide 22
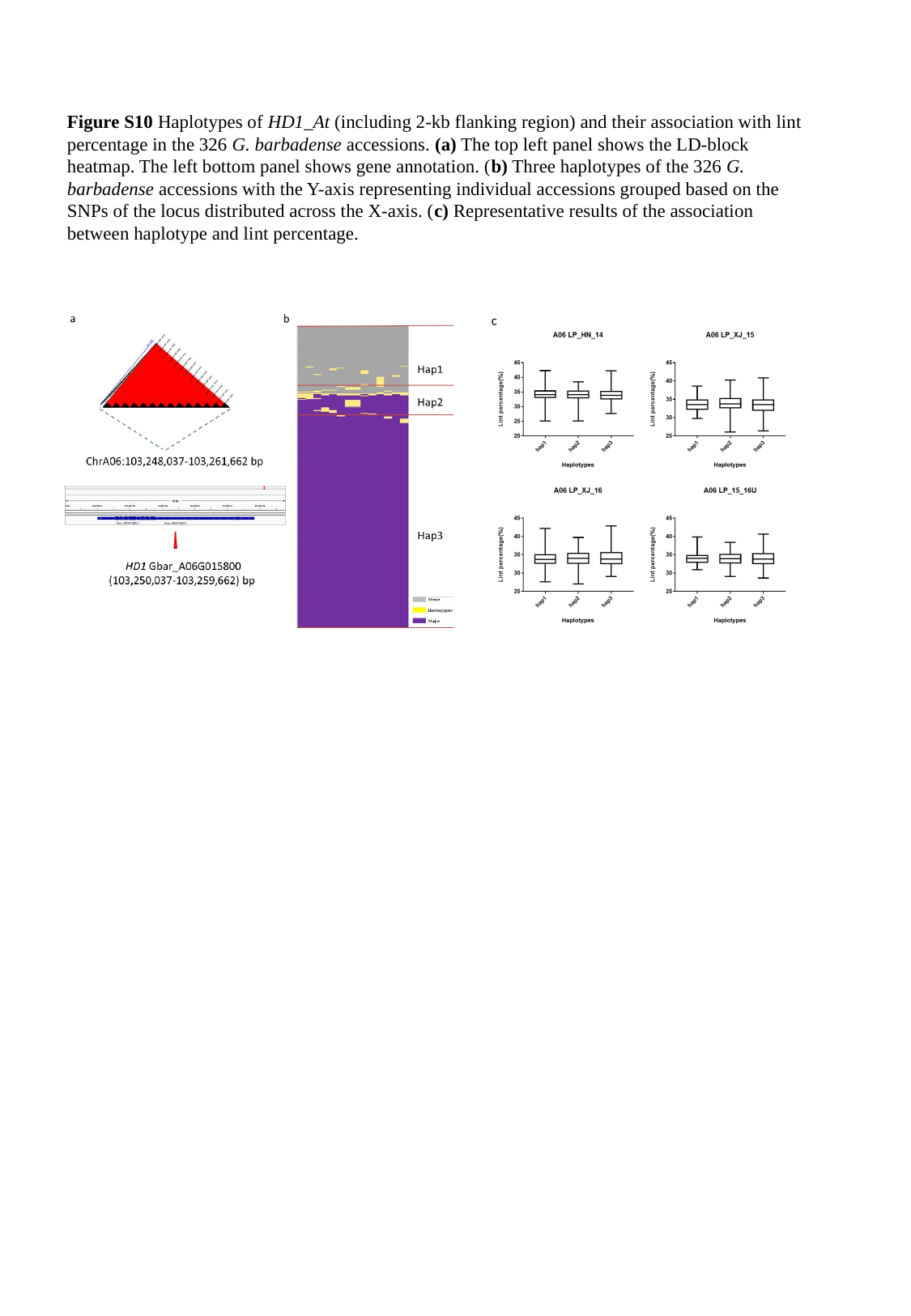

Figure S10 Haplotypes of HD1_At (including 2-kb flanking region) and their association with lint percentage in the 326 G. barbadense accessions. (a) The top left panel shows the LD-block heatmap. The left bottom panel shows gene annotation. (b) Three haplotypes of the 326 G. barbadense accessions with the Y-axis representing individual accessions grouped based on the SNPs of the locus distributed across the X-axis. (c) Representative results of the association between haplotype and lint percentage.
