# Supplementary material for: Dissecting the major genetic components underlying cotton lint development
Source: Genetics. 2023 Dec 26;226(2):iyad219. doi: 10.1093/genetics/iyad219 (PMC10847716; doi:10.1093/genetics/iyad219)
Supplement: iyad219_Supplementary_Data [file iyad219_supplementary_data.zip › Supplemental_Material_Legends_GENETICS-2023-306698.docx]

**Supplementary materials**

**Supplement Figure 1** Seed phenotype of SL1-7-1 under the greenhouse conditions in Canberra, Australia.

**Supplemental Figure 2** SNP frequency (Y-axis) of the low and high lint% pools of SS F_2_ (SL1-7-1 x Sicala V-2) across the 26 cotton chromosomes.

**Supplemental Figure 3** SNP frequency (Y-axis) of the low and high lint% pools of ST F_2_ (SL1-7-1 x T586) across the 26 cotton chromosomes.

**Supplemental Figure 4** The genomic region on A06 containing *HD1_At* was identified to be associated with low lint% in the ST F_2_ population based on BSA.

**Supplemental Figure 5** Schematic diagrams showing the gene structure (not-to-scale) of *MYB25-like_At*, *MYB25-like_Dt*, *HD1_At* and *HD1-Dt*.

**Supplemental Figure 6** SNP frequency (Y-axis) of the low and high lint% pools of TPT BC_1_F_2_ [(T586 x Pima S-7) x T586] across the 26 cotton chromosomes.

**Supplemental Figure 7** The genomic region on A06 containing *HD1_At* was identified to be associated with low lint% in the TPT BC_1_F_2_ population based on BSA.

**Supplemental Figure 8** SNP frequency (Y-axis) of the low and high lint% pools of TPP SBC_1_F_2_ across the 26 cotton chromosomes.

**Supplemental Figure 9** The genomic region on D06 containing *HD1_Dt* was identified to be associated with low lint% in the TPP SBC_1_F_2_ population based on BSA.

**Supplemental Figure 10** Haplotypes of *HD1_At* (including 2-kb flanking region) and their association with lint percentage in the 326 *G. barbadense* accessions.

**Supplementary Table 1** Primers used in the study.

**Supplementary Table 2** Genotypes of the parental lines of the four TP F_2:3_ populations used in assessing the effect of *MYB25-like* and *HD1* on lint percentage.

**Supplementary Table 3** The effect of different combinations of the four loci on lint percentage.

**Supplementary Table 4** The 723 *G. hirsutum* cotton accessions used in this study.

**Supplementary Table 5** The 326 *G. barbadense* cotton accessions used in this study.

**Supplementary File1** The file contains the lint% data, KASP results, seed phenotype of all segregating populations, and the qPCR results used in generating Figure 3.
